# Chromosome-level genome assembly of the shuttles hoppfish, *Periophthalmus modestus*

**DOI:** 10.1093/gigascience/giab089

**Published:** 2022-01-12

**Authors:** Youngik Yang, Ji Yong Yoo, Sang Ho Baek, Ha Yeun Song, Seonmi Jo, Seung-Hyun Jung, Jeong-Hyeon Choi

**Affiliations:** Department of Applied Research, National Marine Biodiversity Institute of Korea, Seocheon 33662, South Korea; Marine Bio-Resources and Information Center, National Marine Biodiversity Institute of Korea, Seocheon 33662, South Korea; Marine Bio-Resources and Information Center, National Marine Biodiversity Institute of Korea, Seocheon 33662, South Korea; Division of Bioresources Bank, Honam National Institute of Biological Resources, Mokpo 58762, South Korea; Department of Applied Research, National Marine Biodiversity Institute of Korea, Seocheon 33662, South Korea; Department of Applied Research, National Marine Biodiversity Institute of Korea, Seocheon 33662, South Korea; Department of Applied Research, National Marine Biodiversity Institute of Korea, Seocheon 33662, South Korea

**Keywords:** shuttles hoppfish, shuttles mudskipper, Periophthalmus modestus, draft genome, PacBio sequencing, Hi-C sequencing

## Abstract

**Background:**

The shuttles hoppfish (mudskipper), *Periophthalmus modestus*, is one of the mudskippers, which are the largest group of amphibious teleost fishes, which are uniquely adapted to live on mudflats. Because mudskippers can survive on land for extended periods by breathing through their skin and through the lining of the mouth and throat, they were evaluated as a model for the evolutionary sea-land transition of Devonian protoamphibians, ancestors of all present tetrapods.

**Results:**

A total of 39.6, 80.2, 52.9, and 33.3 Gb of Illumina, Pacific Biosciences, 10X linked, and Hi-C data, respectively, was assembled into 1,419 scaffolds with an N50 length of 33 Mb and BUSCO score of 96.6%. The assembly covered 117% of the estimated genome size (729 Mb) and included 23 pseudo-chromosomes anchored by a Hi-C contact map, which corresponded to the top 23 longest scaffolds above 20 Mb and close to the estimated one. Of the genome, 43.8% were various repetitive elements such as DNAs, tandem repeats, long interspersed nuclear elements, and simple repeats. *Ab initio* and homology-based gene prediction identified 30,505 genes, of which 94% had homology to the 14 Actinopterygii transcriptomes and 89% and 85% to Pfam familes and InterPro domains, respectively. Comparative genomics with 15 Actinopterygii species identified 59,448 gene families of which 12% were only in *P. modestus*.

**Conclusions:**

We present the high quality of the first genome assembly and gene annotation of the shuttles hoppfish. It will provide a valuable resource for further studies on sea-land transition, bimodal respiration, nitrogen excretion, osmoregulation, thermoregulation, vision, and mechanoreception.

## Introduction

Mudskippers are of the subfamily Oxudercinae and the family Oxudercidae, which was recently separated from the family Gobiidae [1], and the largest group of amphibious teleost fishes, which are uniquely adapted to live on mudflats [2]. They can survive on land for extended periods by breathing through their skin and through the lining of the mouth and throat. They propel themselves over land on their sturdy forefins, and some of them are also able to climb trees and skip atop the surface of the water [3]. They inhabit tropical, subtropical, and temperate regions, including the Indo-Pacific and the Atlantic coast of Africa [4].

The family Oxudercidae has 10 genera and 42 species in FishBase. Among them, 4 species have been sequenced for the draft genome [2]. However, only *Boleophthalmus pectinirostris* is useful as a draft genome.

**Figure 1:**
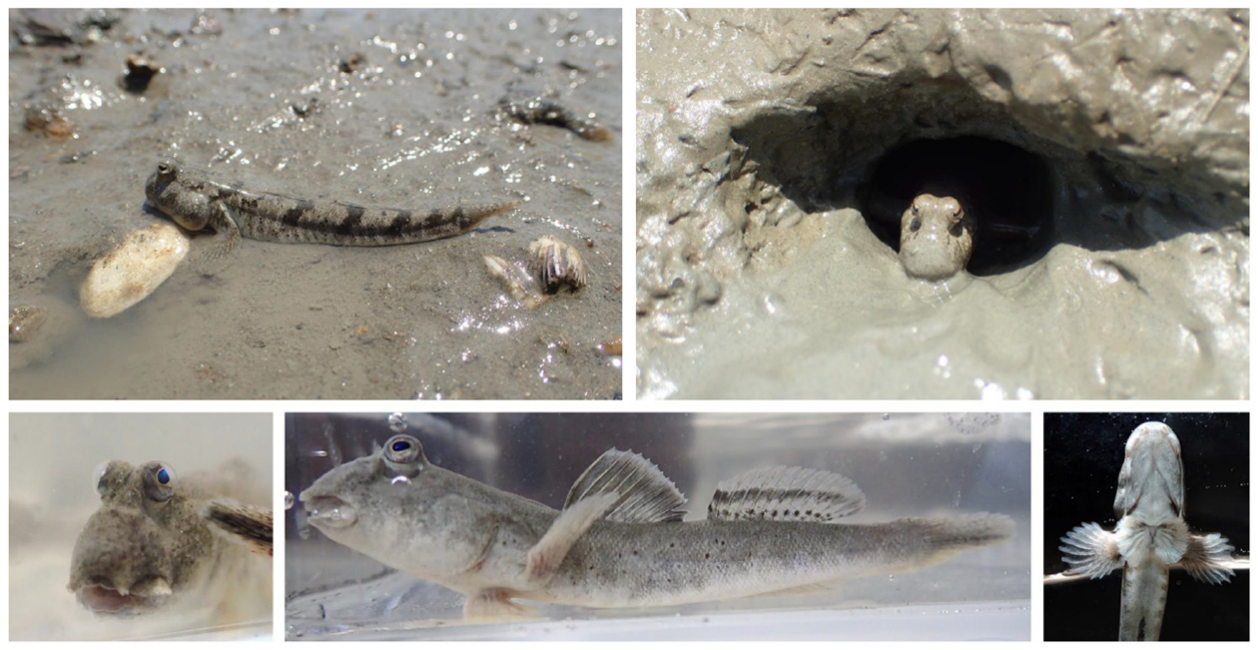
. Adult *Periophthalmus modestus* used in this study. Upper images show the *P. modestus* found in their natural habitat, moving on the surface or hiding in a hole in the tidal flats. Lower images show the frontal, lateral, and ventral view of the specimen, respectively.

In this study, we present a chromosome-level high-quality genome of *Periophthalmus modestus* (NCBI:txid146921; Fishbase ID: 54509) using Pacific Biosciences (PacBio) long-read, Illumina short-read, 10X linked read, and Hi-C sequencing. *P. modestus* [5] is a species of the shuttles hoppfish occurring worldwide in tropical and temperate near shore–marine habitats, including the northwestern Pacific Ocean from Vietnam to Korea, as well as Japan [6]. *P. modestus* can reach a length of 10 cm (Fig. 1) and was known to have 23 chromosomes [7]. We performed structural gene annotation and repeats analysis. Comparative genomics with 16 Actinopterygii genomes identified synteny map, orthologous gene families, evolutionary divergence, and expanded and contracted gene families.

## Methods

### Sample collection and extraction of genomic DNA and total RNA

*P. modestus* samples were collected from Gochang-gun, Jeollabuk-do, South Korea (35 20 24.0 N, 126 22 12.0 E), in May 2018. Total DNA was isolated from the muscle of *P. modestus* using the DNeasy Blood & Tissue kit (Qiagen, Germantown MD USA), following the manufacturer’s protocol.

For species identification, the mitochondrial DNA cytochrome b gene barcode region was amplified using PCR as described in [8]. The PCR product of ∼803 bp was purified using the QIAquick PCR purification kit (Qiagen, Germantown MD, USA) and sequenced on an ABI 3730xl DNA Analyzer (Applied Biosystems 3730xl Capillary Genetic Sequencer, RRID:SCR_018059) with the same PCR primer set. The sequence data were edited and aligned using the ATGC 4.0 software (Genetyx, Japan).

Organs of specimens collected in July 2019 were manually dissected for eye, brain, liver, gut, muscle, and fin tissues, and total RNA was extracted from the dissected organs using the RNeasy Mini Kit (Qiagen, Germantown MD USA). The RNA preparation was repeated 3 times, and then 3-replicate RNA samples were mixed and processed for RNA sequencing (RNA-seq) and isoform sequencing (Iso-seq).

### DNA library construction and sequencing

For short-read sequencing, a paired-end library with insert sizes of 550 bp was constructed using Illumina TruSeq DNA Nano Prep Kit (Illumina, San Diego CA USA) and sequenced on an Illumina HiSeq 4000 instrument (Illumina HiSeq 4000 System, RRID:SCR_016386). For long-read sequencing, a 20-kb SMRTbell library (PacBio, Menlo Park CA USA) was prepared and sequenced on a PacBio Sequel (PacBio Sequel System, RRID:SCR_017989) using 11 cells. To increase continuity in the genome assembly, we further produced linked reads and Hi-C reads. For linked-read sequencing, a 10X Chromium genome v2 library (10X Genomics, Pleasanton CA USA) was constructed and sequenced on an Illumna NovaSeq 6000 instrument. For long-range scaffolding, a Dovetail Hi-C library was prepared with Dovetail Hi-C Library kit (Dovetail Genomics, Scotts Valley CA USA) and sequenced on an Illumina NovaSeq 6000 instrument (Illumina NovaSeq 6000 Sequencing System, RRID:SCR_016387).

### RNA library construction and sequencing

For RNA-seq, paired-end libraries with insert size of 150 bp were prepared with the Truseq mRNA Prep kit (Illumina, San Diego CA USA) from total messenger RNA (mRNA), which was subsequently sequenced on an Illumina HiSeq 2500 (Illumina HiSeq 2500 System, RRID:SCR_016383). For PacBio Iso-seq, 3 libraries of length 1–2, 2–3, and 3–6 kb were prepared from polyadenylated RNA according to the PacBio Iso-seq protocol (PacBio, Menlo Park CA USA). Six SMRT cells were run on a PacBio RS II system (PacBio RS II Sequencing System, RRID:SCR_017988).

### Genome size estimation

Trimmomatic (Trimmomatic, RRID:SCR_011848) [9] was used to clean raw short reads by removing leading and trailing low-quality regions or those that contained the TruSeq index and universal adapters. JELLYFISH (Jellyfish, RRID:SCR_005491) [10] generated a 17-mer distribution and GenomeScope (GenomeScope, RRID:SCR_017014) [11] estimated the size where the main peak was chosen.

### Genome assembly and evaluation

MiniASM [12] assembled contigs from pairwise alignments generated by MiniMap2 (Minimap2, RRID:SCR_018550) [13] using PacBio long reads. Contigs were polished using RACON (Racon, RRID:SCR_017642) [14] with the alignments generated by MiniMap2 (Minimap2, RRID:SCR_018550) using PacBio long reads, and further polished using Pilon (Pilon, RRID:SCR_014731) [15] with the alignments generated by BWA (BWA, RRID:SCR_010910) [16] using Illumina short reads. Then, 10X Genomics linked reads were used to correct misassembled contigs using tigmint [17] and to generate scaffolds using ARCS [18] and LINKS [19]. Dovetail HiRise assembler [20] linked the scaffolds to pseudo-chromosomes. In brief, Hi-C reads were aligned to the scaffolds using a modified version of SNAP (SNAP, RRID:SCR_007936) and PCR duplicates were marked using Novosort [20]. Then HiRise analyzed the separations of Hi-C read pairs mapped within the scaffolds to produce a likelihood model for the genomic distance between read pairs, and the model was used to identify and break putative misjoins, to score prospective joins, and to make joins above a threshold. QUAST (QUAST, RRID:SCR_001228) [21] accessed the length statistics of the genome assembly, and BUSCO (BUSCO, RRID:SCR_015008) [22] evaluated the completeness of genome and transcriptome with metazoa conserved genes. Purge_dups (purge dups, RRID:SCR_021173) [23] purged haplotigs and heterozygous overlaps.

### Repeat analysis

Repeats were predicted in 3 ways. Tandem Repeats Finder [24] identified tandem repeats. RepeatMasker (RepeatMasker, RRID:SCR_012954) [25] identified transposable elements with a *de novo* library built by RepeatModeler (RepeatModeler, RRID:SCR_015027) [26] and with a known library (Fugu) in RepBase (Repbase, RRID:SCR_021169) [27] using RMBlast.

### Gene prediction and annotation

We combined *de novo*, RNA-based and homology-based methods to carry out protein-coding gene prediction. For the *de novo* and RNA-based gene prediction, Illumina RNA-seq and PacBio Iso-seq datasets were used to generate 2 hint files. Tophat (TopHat, RRID:SCR_013035) [28] aligned RNA-seq reads to the soft repeat-masked genome assembly. To obtain intron hints from Iso-seq, LSC [29] corrected sequencing errors in full-length transcripts with RNA-seq, GMAP (GMAP, RRID:SCR_008992) [30] aligned the corrected transcripts to the genome, and gmap2hints.pl in the AUGUSTUS package (Augustus, RRID:SCR_008417) [31] generated intron hints from the alignments. BRAKER (BRAKER, RRID:SCR_018964) [32] predicted protein-coding genes by incorporating the outputs of GeneMark-ET (GeneMarker, RRID:SCR_015661) [33] and AUGUSTUS (Augustus, RRID:SCR_008417). GeneMark-ET (GeneMarker, RRID:SCR_015661) predicts genes with unsupervised training, whereas AUGUSTUS (Augustus, RRID:SCR_008417) predicts genes with supervised training based on intron and protein hints.

For the homology-based gene prediction, the assembly of *P. modestus* was aligned against the genes of 14 Actinopterygii genomes (Supplementary Table S1) and vertebrata in orthoDB (OrthoDB, RRID:SCR_011980) using TBLASTN (TBLASTN, RRID:SCR_011822) [34] with an E-value cut-off of 1E−5. GenBlastA (genBlastA, RRID:SCR_020951) [35] clustered matching sequences and retained only the best-matched regions, which were used to predict gene models for a homology-based approach using Exonerate (Exonerate, RRID:SCR_016088) [36]. Finally, the homology-based gene prediction was merged to the *ab initio* prediction only when there was no conflict. Then the merged genes were removed if their coding sequences contained premature stop codons or were not supported by hints. InterProScan (InterProScan, RRID:SCR_005829) [37] annotated the predicted genes with various databases, including Hamap (HAMAP, RRID:SCR_007701) [38], Pfam (Pfam, RRID:SCR_004726) [39], PIRSF (PIRSF, RRID:SCR_003352) [40], PRINTS (PRINTS, RRID:SCR_003412) [41], ProDom (ProDom, RRID:SCR_006969) [42], PROSITE (PROSITE, RRID:SCR_003457) [43], SUPERFAMILY (SUPERFAMILY, RRID:SCR_007952) [44], and TIGRFAMS (TIGRFAMS, RRID:SCR_005493) [45].

To predict non-coding genes, Infernal (Infernal, RRID:SCR_011809) [46], RNAmmer (RNAmmer, RRID:SCR_017075) [47], and tRNAscan (tRNAscan-SE, RRID:SCR_010835) [48] were used.

### Comparative genomics

Chromeister [49] performed all pairwise comparison with 17 Actinopterygii genomes to generate a synteny map. OrthoMCL (OrthoMCL DB: Ortholog Groups of Protein Sequences, RRID:SCR_007839) [50] identified orthologous gene families among 15 Actinopterygii transcriptomes (Supplementary Table S1). GO (Gene Ontology, RRID:SCR_002811) enrichment was performed using the Fisher exact test and false discovery rate correction to identify functionally enriched GO terms among gene families relative to the “genome background,” as annotated by Pfam.

For phylogenetic analysis and divergence time estimation, MUSCLE (MUSCLE, RRID:SCR_011812) [51] aligned the amino acid sequences of single-copy gene families, trimAl (trimAl, RRID:SCR_017334) [52] filtered low alignment quality regions, RAxML (RAxML, RRID:SCR_006086) [53] constructed a phylogenetic tree with the PROTGAMMAJTT model (100 bootstrap replicates), and MEGA7 (MEGA Software, RRID:SCR_000667) [54] calculated divergence time with the Jones–Taylor–Thornton model and the previously determined topology. Gene family expansion and contraction were analyzed by CAFE (CAFE, RRID:SCR_005983) [55] with the identified orthologous gene families and the estimated phylogenetic information. Supplementary Table S12 shows the software versions, settings, and parameters.

## Results

### Species identification

Comparison of cytochrome b sequences against the NCBI GenBank database (http://www.ncbi.nlm.nih.gov/) showed >99% sequence identity to *P. modestus* (GenBank accession No. DQ901364.1), 89% to *Periophthalmus argentilineatus* (AP019359.1), and 85% to *Periophthalmus barbarus* (KF415633.1).

### Chromosome-level genome assembly

We generated 39.6, 80.2, 52.9, and 33.3 Gb (46×, 94×, 62×, and 39× coverage) of Illumina, PacBio, 10X linked, and Hi-C data, respectively, for genome sequencing (Supplementary Table S2). The genome size was estimated at 729 Mb using the 17-mer peak and distribution from cleaned Illumina data (Supplementary Fig. S1). MiniMAP2 and MiniASM followed by polishing using RACON and Pilon generated 3,839 contigs (854 Mb and N50 of 579 kb) using PacBio sequencing data. Tigmint, ARCS, and LINKS generated 2,170 scaffolds (854 Mb and N50 of 1.5 Mb) using 10X linked data, and Dovetail HiRise finally generated 1,419 scaffolds including 23 pseudo-chromosomes (854 Mb and N50 of 33 Mb) using Hi-C data (Table 1). The pseudo-chromosomes were anchored by a Hi-C contact map (Supplementary Fig. S2), and corresponded to the top 23 longest scaffolds, of which the sum of lengths was close to the estimated genome size (742 Mb, Supplementary Table S3). Interestingly, the number of pseudo-chromosomes is the same as that of chromosomes [7]. Table 1 presents the length statistics of the genome assembly, while Supplementary Table S4 reports the genome completeness of 96.3% for contigs and scaffolds. Haplotigs and heterozygous overlaps of length 45 Mb were purged, leaving 665 scaffolds (810 Mb and N50 of 32.9 Mb).

**Table 1.** Statistics of the genome assembly

	Contigs	Scaffolds
No. contigs (≥0 bp)	3,839	1,419
No. contigs (≥10,000 bp)	3,828	1,370
No. contigs (≥50,000 bp)	2,784	581
Total length (≥0 bp)	854,179,206	854,451,706
Total length (≥10,000 bp)	854,103,429	854,168,706
Total length (≥50,000 bp)	818,910,422	829,641,531
No. contigs	3,839	1,419
Largest contig	5,687,114	44,673,496
Total length	854,179,206	854,451,706
GC (%)	40.64	40.64
N50	579,133	32,909,307
N75	227,794	28,196,589
L50	375	12
L75	953	19
Nucleotides per 100 kb	0	31.89

### Genome annotation

**Figure 2:**
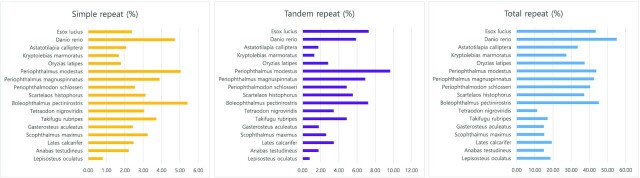
. Percentage of the genome for simple, tandem, and total repeats for 17 Actinopterygii species.

Repetitive elements predicted by the 3 ways were merged to a total of 452 Mb, which covered 44% of the genome: 11, 6, 5, 10, and 17% for DNA, long interspersed nuclear elements, simple repeats, tandem repeats, and unknown, respectively (Supplementary Table S5). We compared *P. modestus* with 16 Actinopterygii species for repeats (Supplementary Table S7). As shown in Fig. 2, *P. modestus* had more simple and tandem repeats than the other Actinopterygii species.

For *ab initio* gene prediction, we generated 172 Gb and 125 Mb of RNA-seq and PacBio data, respectively, which yielded 366,298 and 131,807 hints for introns. BRAKER with GeneMark and AUGUSTUS predicted 132,821 genes. For homology-based gene prediction, we used 14 Actinopterygii species (Supplementary Table S1). A pipeline of TBLASTN, GenBlastA, and Exonerate predicted 22,721 genes. Merging the 2 outputs and filtering incomplete genes produced 30,505 genes and 34,916 transcripts (Supplementary Table S6), of which 94% had homology to the 14 Actinopterygii transcriptomes. As a result of InterProScan annotation, 27,048 genes had 5,489 Pfam families, 25,995 genes had 5,121 InterPro domains, 17,310 genes had 2,277 GO terms, and 6,059 genes had 2,166 pathways.

Infernal predicted 5,071 non-coding genes such as long non-coding RNA, microRNA, and miscellaneous RNA, while tRNAscan predicted 4,510 transfer RNAs (tRNAs) with 25 types (Supplementary Table S11). RNAmmer predicted 1,950 ribosomal RNAs (rRNAs): 1,836, 53, and 61 for 8s, 18s, and 28s rRNA, respectively.

### Synteny map

The 17 Actinopterygii genomes (Supplementary Table S1) were compared to identify a synteny map using Chromeister. Supplementary Fig. S3 shows dot plots in the upper triangular matrix and distance scores in the lower triangular matrix. As expected, the pair of *P. modestus* and *Periophthalmus magnuspinnatus* had the lowest score, meaning the closest pair. The second and third lowest score corresponded to the pair of *Boleophthalmus pectinirostris* with *P. magnuspinnatus* and *P. modestus*, respectively. Note that the scores of *Danio rerio* and *Lepisosteus oculatus* with the others were >0.99 because of the evolutionary distances.

### Orthologous gene family

**Figure 3:**
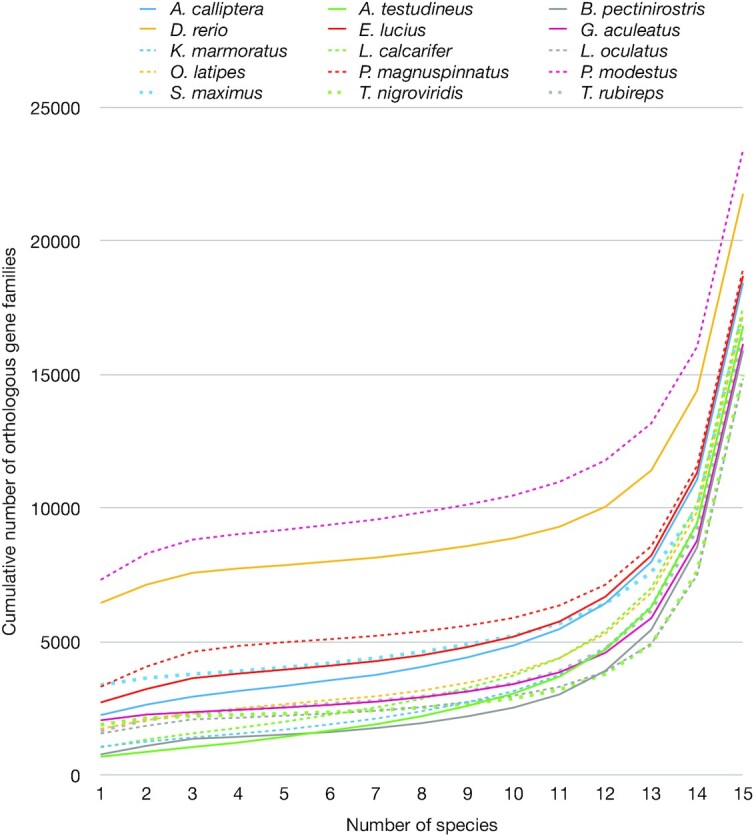
Cumulative number of orthologous gene families per the number of species with regard to a specified species.

The 15 Actinopterygii whole-genome gene datasets (Supplementary Table S1) were compared to identify orthologous gene families using orthoMCL. Among 59,448 gene families, 7,358 were common in all genomes, while 2,265, 707, 792, 6,461, 2,737, 2,070, 1,082, 1,059, 1,576, 1,751, 3,326, 7,326, 3,389, 1,901, and 1,686 were only in *Astatotilapia calliptera, Anabas testudineus, B. pectinirostris, D. rerio, Esox lucius, Gastersteus aculeatus, Kryptolebias marmoratus, Lates calcarifer, L. oculatus, Oryzias latipes, P. magnuspinnatus, P. modestus, Scophthalmus maximus, Tetraodon nigroviridis*, and *Takifugu rubireps*, respectively. As shown in Fig. 3, *P. modestus* had more families than the others and the number of common families in ≥13 species were dominant. The unique gene families of *P. modestus* were enriched in negative regulation of RNA metabolic and biosynthetic process, nucleic acid-templated, transcription DNA-templated, nucleobase-containing, biosynthetic process, and cellular macromolecule (Supplementary Table S8).

### Phylogenetic relationships and divergence time

**Figure 4:**
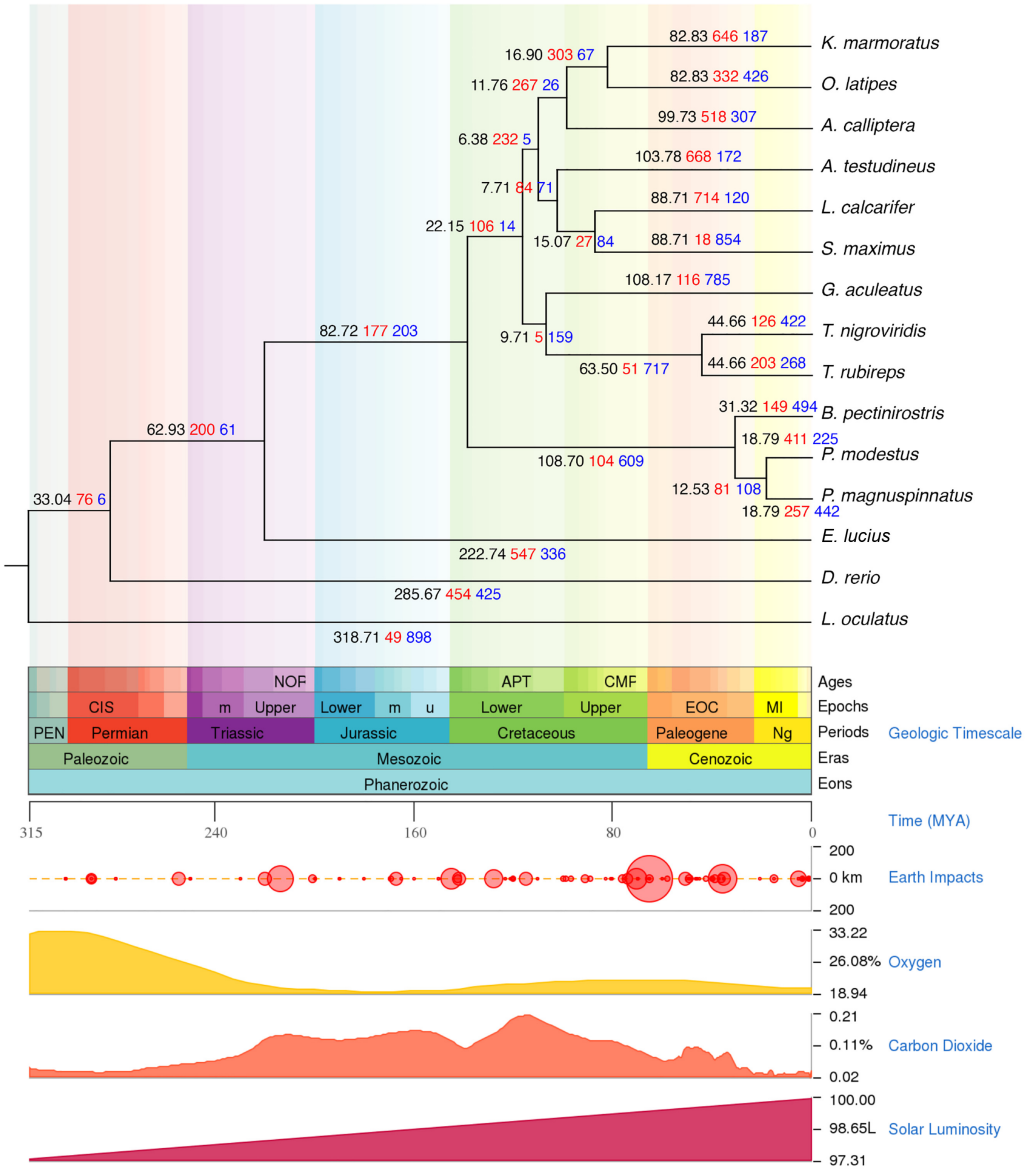
. Time tree was constructed by MEGA7 with 281 single-copy orthologous gene families among 15 Actinopterygii, where the first (black) numbers represent divergence time in millions of years (MYA); the second (red) and third (blue) numbers represent the number of expanded and contracted, respectively, gene families identified by CAFE; the geologic timescale, earth impacts, oxgen, carbon dioxide, and solar luminosity were generated on the TimeTree database.

All genomes had 281 single-copy orthologous gene families, which were used to construct a phylogenetic tree and estimate divergence time. The TimeTree database [56] was used to take calibration times between *L. calcarifer*–*S. maximus, K. marmoratus*–*O. latipes*, and *T. rubireps*–*T. nigroviridis* divergence as 70–94, 76–114, and 42–59 MYA. As shown in Fig. 4, the infraclass Teleostei was separated at ~320 MYA, consistent with the previous study [57], the order Cypriniformes at ~287 MYA, the order Esociformes at ~224 MYA, and the order Gobiiformes at ~141 MYA. *P. modestus* clustered with the other species in the order Gobiiformes, and diverged from *P. magnuspinnatus* and *B. pectinirostris* during the late and mid-Cenozoic era (15 and 25 MYA), respectively.

### Gene family expansion and contraction

Orthologous gene families among the 15 Actinopterygii genomes were used for analyzing gene family expansion and contraction. The number of expanded and contracted gene families of *P. modestus* with its common ancestor were 411 and 225, while those of *P. magnuspinnatus*, the closest genome, were 257 and 442, respectively (Fig. 4). The expanded gene families of *P. modestus* were enriched in base-excision repair, transmembrane receptor protein tyrosine kinase signaling pathway, and enzyme linked receptor protein signaling pathway (Supplementary Table S9), while the contracted gene families of *P. modestus* were in FMN binding, ion binding, and reactive oxygen species metabolic process (Supplementary Table S10). Supplementary Fig. S4 shows a word cloud for GO term description enriched in unique, expanded, and contracted gene families of *P. modestus*.

## Conclusions

We present a chromosome-level high-quality genome assembly of *P. modestus* with N50 length of 33 Mb using Illumina, PacBio, 10X, Hi-C, RNA, and Isoform sequencing, respectively. The completeness of the genome was confirmed by the BUSCO score of 96.3%. The top 23 longest scaffolds were >20 Mb in size and close to the estimated genome size of 728 Mb. *P. modestus* had various repetitive elements in 43.8% of the genome and more repetitive elements than the 16 Actinopterygii genomes. We predicted 34,871 protein-coding and 7,865 non-coding genes, and 93% of the protein-coding genes had homology to the 14 Actinopterygii transcriptomes. This dataset will provide a valuable resource for further studies on sea-land transition, bimodal respiration, nitrogen excretion, osmoregulation, thermoregulation, vision, and mechanoreception.

## Data Availability

All raw sequencing reads underlying this article are available in the NCBI SRA (Supplementary Table S2) and can be accessed with BioProject No. PRJNA660579. The assembled genome was submitted to NCBI Assembly. Gene annotation and transcript sequences are provided as supplementary files. JBrowse [58] was set up on http://magic.re.kr/gbrowser/jb/mabik/?data=shuttles_hoppfish. All supporting data and materials are available in the *GigaScience* GigaDB database [59].

## Additional Files

**Supplementary Figure S1**. Genome size estimation by 17-mer distribution.

**Supplementary Figure S2**. Hi-C contact map.

**Supplementary Figure S3**. Synteny map of 17 Actinopterygii genomes.

**Supplementary Figure S4**. Word cloud for GO term description.

**Supplementary Table S1**. Taxonomy and statistics of 17 Actinopterygii species.

**Supplementary Table S2**. Statistics of sequencing data.

**Supplementary Table S3**. Top 23 longest scaffolds.

**Supplementary Table S4**. BUSCO assessment of genome assembly and gene prediction with metazoa.

**Supplementary Table S5**. Statistics of repetitive elements.

**Supplementary Table S6**. Statistics of predicted protein-coding genes.

**Supplementary Table S7**. Repeat analysis for the 17 Actinopterygii genome.

**Supplementary Table S8**. Top 40 GO terms enriched in unique gene families of *P. modestus*.

**Supplementary Table S9**. Top 40 GO terms enriched in expanded gene families of *P. modestus*.

**Supplementary Table S10**. Top 40 GO terms enriched in contracted gene families of *P. modestus*.

**Supplementary Table S11**. Statistics of predicted non-coding genes.

**Supplementary Table S12**. A list of software and parameters used for genome analyses.

## Abbreviations

bp: base pairs; BUSCO: Benchmarking Universal Single-Copy Orthologs; BWA: Burrows-Wheeler Aligner; Gb: gigabase pairs; GO: gene ontology; Iso-seq: Isoform sequencing; kb: kilobase pairs; Mb: megabase pairs; MYA: million years ago; NCBI: National Center for Biotechnology Information; PacBio: Pacific Biosciences; RAxML: Randomized Axelerated Maximum Likelihood; RNA-seq: RNA sequencing; rRNA: ribosomal RNA; SMRT: single-molecule real-time; SNAP: Scalable Nucleotide Alignment Program; SRA: Sequence Read Archive; tRNA: transfer RNA.

## Competing Interests

The authors declare that they have no competing interests.

## Funding

This study was financially supported by the National Marine Biodiversity Institute of Korea Research Program (2021M00600).

## Authors' Contributions

J.H.C. and Y.Y. conceived the concept; H.Y.S., S.J., and S.H.J. collected and classified the sample; J.H.C. and Y.Y. designed the experiments; J.Y.Y., S.H.B., Y.Y., and J.H.C. analyzed the genomic data; S.H.B. and Y.Y. deposited the data into NCBI; and H.Y.S., Y.Y., and J.H.C. wrote the manuscript. All authors reviewed the manuscript.

## Supplementary Material

giab089_GIGA-D-21-00233_Original_Submission

giab089_GIGA-D-21-00233_Revision_1

giab089_GIGA-D-21-00233_Revision_2

giab089_GIGA-D-21-00233_Revision_3

giab089_Response_to_Reviewer_Comments_Revision_2

giab089_Reviewer_1_Report_Original_Submission Jiang Hu -- 9/1/2021 Reviewed

giab089_Reviewer_2_Report_Original_Submission Yunyun Lv -- 9/7/2021 Reviewed

giab089_Reviewer_2_Report_Revision_1 Yunyun Lv -- 11/9/2021 Reviewed

giab089_Supplemental_Figures_and_Tables
