# Supplementary material for: Chromosome-level genome assembly of the shuttles hoppfish, Periophthalmus modestus
Source: Gigascience. 2022 Jan 12;11:giab089. doi: 10.1093/gigascience/giab089 (PMC8756193; doi:10.1093/gigascience/giab089)
Supplement: giab089_GIGA-D-21-00233_Revision_2 [file giab089_giga-d-21-00233_revision_2.pdf]

GigaScience

Chromosome-level genome assembly of the shuttles hoppfish, Periophthalmus modestus

--Manuscript Draft--

**Manuscript Number:**	GIGA-D-21-00233R2
**Full Title:**	Chromosome-level genome assembly of the shuttles hoppfish, Periophthalmus modestus
**Article Type:**	Data Note
**Funding Information:**	national marine biodiversity institute of korea (2021M00600) — Not applicable
**Abstract:**	Background The shuttles hoppfish (mudskipper), Periophthalmus modestus , is one of mudskippers which are the largest group of amphibious teleost fishes that are uniquely adapted to live on mudflats. Since mudskippers can survive on land for extended periods of time by breathing through their skin and through the lining of the mouth and throat, they were evaluated as a model for the evolutionary sea-land transition of Devonian protoamphibians, ancestors of all present tetrapods.<br><br>Results A total of 39.6, 80.2 and 52.9 Gbp of Illumina, PacBio, 10X linked and Hi-C data, respectively, was assembled into 1,419 scaffolds with a N50 length of 33 Mbp and BUSCO score of 96.6%. The assembly covered 117% of the estimated genome size (729 Mbp) and included 23 pseudo-chromosomes anchored by a Hi-C contact map, which corresponded to the top 23 longest scaffolds were above 20 Mbp in size and close to the estimated one. Of the genome, 43.8% were various repetitive elements such as DNAs, tandem repeats, LINEs and simple repeats. Ab initio and homology-based gene prediction identified 30,505 genes, of which 94% had homology to the 14 Actinopterygii transcriptomes and 89% and 85% did Pfam familes and InterPro domains respectively. Comparative genomics with 15 Actinopterygii species identified 59,448 gene families of which 12% were only in P. modestus .<br><br>Conclusions We present the high quality of the first genome assembly and gene annotation of the shuttles hoppfish. It will provide a valuable resource for further studies on sea-land transition, bimodal respiration, nitrogen excretion, osmoregulation, thermoregulation, vision and mechanoreception.
**Corresponding Author:**	Jeong-Hyeon Choi, Ph.D.<br>National Marine Biodiversity Institute of Korea<br>Seocheon-gun, Chungcheongnam-do KOREA, REPUBLIC OF
**Corresponding Author Secondary Information:**	
**Corresponding Author's Institution:**	National Marine Biodiversity Institute of Korea
**Corresponding Author's Secondary Institution:**	
**First Author:**	Youngik Yang
**First Author Secondary Information:**	
**Order of Authors:**	Youngik Yang<br>Ji Yong Yoo<br>Sang Ho Baek<br>Ha Yeun Song<br>Seonmi Jo<br>Seung-Hyun Jung<br>Jeong-Hyeon Choi, Ph.D.

Powered by Editorial Manager® and ProduXion Manager® from Aries Systems Corporation

Order of Authors Secondary Information:	
Response to Reviewers:	We uploaded a fie for response.
Additional Information:	
Question	Response
Are you submitting this manuscript to a special series or article collection?	No
**Experimental design and statistics**<br><br>Full details of the experimental design and statistical methods used should be given in the Methods section, as detailed in our Minimum Standards Reporting Checklist. Information essential to interpreting the data presented should be made available in the figure legends.<br><br>Have you included all the information requested in your manuscript?	Yes
**Resources**<br><br>A description of all resources used, including antibodies, cell lines, animals and software tools, with enough information to allow them to be uniquely identified, should be included in the Methods section. Authors are strongly encouraged to cite Research Resource Identifiers (RRIDs) for antibodies, model organisms and tools, where possible.<br><br>Have you included the information requested as detailed in our Minimum Standards Reporting Checklist?	Yes
**Availability of data and materials**<br><br>All datasets and code on which the conclusions of the paper rely must be either included in your submission or deposited in publicly available repositories (where available and ethically appropriate), referencing such data using a unique identifier in the references and in	Yes

Powered by Editorial Manager® and ProduXion Manager® from Aries Systems Corporation

the “Availability of Data and Materials” section of your manuscript.<br><br>Have you have met the above requirement as detailed in our Minimum Standards Reporting Checklist?	

Powered by Editorial Manager® and ProduXion Manager® from Aries Systems Corporation

Manuscript

Click here to access/download;Manuscript;mudskipper.pdf

Placeholder for OUP logo oup.pdf

GigaScience, 0000, 1–7

doi: xx.xxxx/xxxx
Manuscript in Preparation
Data note

DATA NOTE

Chromosome-level genome assembly of the shuttles hoppfish, *Periophthalmus modestus*

Youngik Yang[1], Ji Yong Yoo[2], Sang Ho Baek[2], Ha Yeun Song[3], Seonmi Jo[1], Seung-Hyun Jung[1] and Jeong-Hyeon Choi[1,*]

[1]Department of Applied Research and [2]Marine Bio-Resources and Information Center, National Marine Biodiversity Institute of Korea, Seocheon, 33662, South Korea, and [3]Division of Bioresources Bank, Honam National Institute of Biological Resources, Mokpo, 58762, South Korea

*jeochoi@gmail.com

Abstract

Background
The shuttles hoppfish (mudskipper), *Periophthalmus modestus*, is one of mudskippers which are the largest group of amphibious teleost fishes that are uniquely adapted to live on mudflats. Since mudskippers can survive on land for extended periods of time by breathing through their skin and through the lining of the mouth and throat, they were evaluated as a model for the evolutionary sea-land transition of Devonian protoamphibians, ancestors of all present tetrapods.
Results
A total of 39.6, 80.2, 52.9 and 33.3 Gbp of Illumina, PacBio, 10X linked and Hi-C data, respectively, was assembled into 1,419 scaffolds with a N50 length of 33 Mbp and BUSCO score of 96.6%. The assembly covered 117% of the estimated genome size (729 Mbp) and included 23 pseudo-chromosomes anchored by a Hi-C contact map, which corresponded to the top 23 longest scaffolds above 20 Mbp and close to the estimated one. Of the genome, 43.8% were various repetitive elements such as DNAs, tandem repeats, LINEs and simple repeats. *Ab initio* and homology-based gene prediction identified 30,505 genes, of which 94% had homology to the 14 Actinopterygii transcriptomes and 89% and 85% did Pfam familes and InterPro domains respectively. Comparative genomics with 15 Actinopterygii species identified 59,448 gene families of which 12% were only in *P. modestus*.
Conclusions
We present the high quality of the first genome assembly and gene annotation of the shuttles hoppfish. It will provide a valuable resource for further studies on sea-land transition, bimodal respiration, nitrogen excretion, osmoregulation, thermoregulation, vision and mechanoreception.

Key words: shuttles hoppfish; shuttles mudskipper; Periophthalmus modestus; draft genome; PacBio sequencing; Hi-C sequencing

Introduction

Mudskippers are of the subfamily Oxudercinae and the family Oxudercidae which was recently separated from the family Gobiidae (Nelson et al.; 2016), and the largest group of amphibious teleost fishes that are uniquely adapted to live on mudflats (You et al.; 2014). They can survive on land for extended periods of time by breathing through their skin and through the lining of the mouth and throat. They propel themselves over land on their sturdy fore fins, and some of them are also able to climb trees and skip atop the surface of the water (Wicaksono et al.; 2020). They inhibit in tropical, subtropical, and temper-

Compiled on: November 15, 2021.
Draft manuscript prepared by the author.

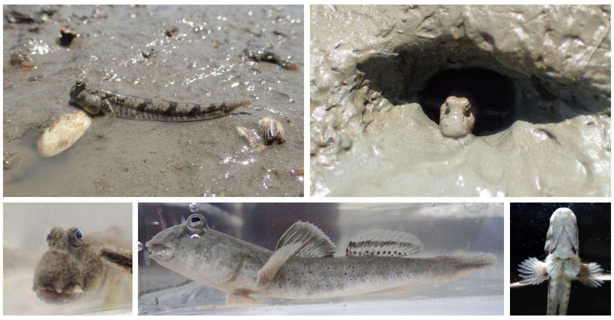

Figure 1. Adult *Periophthalmus modestus* used in this study. Upper images showed the *P. modestus* found in their natural habitat, moving on the surface or hiding in a hole of tidal flats. Lower images showed the frontal, lateral, and ventral view of the specimen, respectively.

ate regions, including the Indo-Pacific and the Atlantic coast of Africa (Parenti and Jaafar; 2017).

The family Oxudercidae has 10 genera and 42 species in FishBase. Among them, four species has been sequenced for the draft genome (You et al.; 2014). However, only *Boleophthalmus pectinirostris* is useful as a draft genome.

In this study, we present a chromosome-level high-quality genome of *Periophthalmus modestus* using PacBio long read, Illumina short read, 10X linked read and Hi-C sequencing. *P. modestus* (Cantor; 1842) is a species of the shuttles hoppfish spread worldwide in tropical and temperate near shore-marine, including the northwestern Pacific Ocean from Vietnam to Korea as well as Japan (Thacker and Roje; 2011). *P. modestus* can reach a length of 10 centimeters (Fig. 1) and was known to have 23 chromosomes (Lee; 1986). We performed structural gene annotation and repeats analysis. Comparative genomics with 16 Actinopterygii genomes identified synteny map, orthologous gene families, evolutionary divergence and expanded and contracted gene families.

Methods

Sample collection and extraction of genomic DNA and total RNA

P. modestus samples were collected from Gochang-gun, Jeollabuk-do, South Korea (35.34N, 126.37E) in May 2018. Total DNA was isolated from the muscle of *P. modestus* using the DNeasy Blood & Tissue kit (QIAGEN, USA), following the manufacturer's protocol.

For species identification, the mitochondrial DNA Cytb gene barcode region was amplified using PCR as described in Chen et al. (2015). The PCR product of approximately 803 bp was purified using the QIAquick PCR purification kit (QIAGEN, USA) and sequenced on an ABI 3730xl DNA Analyzer (Applied Biosystems, USA) with the same PCR primer set. The sequence data were edited and aligned using the ATGC 4.0 software (Genetyx, Japan).

Organs of specimens collected in July 2019 were manually dissected for eye, brain, liver, gut, muscle and fin tissues, and total RNA was extracted from the dissected organs using the RNeasy Mini Kit (Qiagen, USA). The RNA preparation was repeated three times, and then three-replicate RNA samples were mixed and processed for RNA-seq and Iso-seq.

DNA library construction and sequencing

For short read sequencing, a paired-end library with insert sizes of 550 bp was constructed using Illumina TruSeq DNA Nano Prep. Kit (Illumina, USA) and sequenced on an Illumina HiSeq 4000 instrument. For long read sequencing, a 20 kb SMRTbell library (PacBio, USA) was prepared and sequenced on a PacBio Sequel using 11 cells. To increase continuity in genome assembly, we further produced linked reads and Hi-C reads. For linked read sequencing, a 10x Chromium genome v2 library (10X genomics, USA) was constructed and sequenced on an Illumna NovaSeq 6000 instrument. For long range scaffolding, a Dovetail Hi-C library was prepared with Dovetail Hi-C Library kit (Dovetail, USA) and sequenced on an Illumina NovaSeq 6000 instrument.

RNA library construction and sequencing

For RNA-seq, paired-end libraries with insert size of 150 bp were prepared with the Truseq mRNA Prep kit (Illumina, USA) from total mRNA, which was subsequently sequenced on an Illumina HiSeq 2500. For PacBio Iso-seq, three libraries of length 1-2, 2-3 and 3-6 Kbp were prepared from polyA+ RNA according to the PacBio Iso-seq protocol (PacBio, USA). Six SMRT cells were run on a PacBio RS II system.

Genome size estimation

Trimmomatic (Bolger et al.; 2014) was used to clean raw short reads by removing leading and trailing low-quality regions or those that contained the TruSeq index and universal adapters. JELLYFISH (Marçais and Kingsford; 2011) generated a 17-mer distribution and GenomeScope (Vurture et al.; 2017) estimated the size where the main peak was chosen.

Genome assembly and evaluation

MiniASM (Li; 2016) assembled contigs from pairwise alignments generated by MiniMap2 (Li; 2018) using PacBio long reads. Contigs were polished using RACON (Vaser et al.; 2017) with the alignments generated by MiniMap2 using PacBio long reads, and further polished using Pilon (Walker et al.; 2014) with the alignments generated by BWA (Li and Durbin; 2009) using Illumina short reads. Then, 10x Genomics linked reads were used to correct mis-assembled contigs using tigmint (Jackman et al.; 2018) and to generate scaffolds using ARCS (Yeo et al.; 2017) and LINKS (Warren et al.; 2015). Dovetail HiRise assembler (Putnam et al.; 2016) linked the scaffolds to pseudo-chromosomes. In brief, Hi-C reads were aligned to the scaffolds using a modified version of SNAP and PCR duplicates were marked using Novosort (Putnam et al.; 2016). Then HiRise analyzed the separations of Hi-C read pairs mapped within the scaffolds to produce a likelihood model for the genomic distance between read pairs, and the model was used to identify and break putative misjoins, to score prospective joins, and to make joins above a threshold. QUAST (Gurevich et al.; 2013) accessed the length statistics of the genome assembly, and BUSCO (Simão et al.; 2015) evaluated the completeness of genome and transcriptome with metazoa conserved genes. Purged_dups (Guan et al.; 2020) purged haplotigs and heterozygous overlaps.

Repeat analysis

Repeats were predicted by three ways. Tandem Repeat Finder (Benson; 1999) identified tandem repeats. Repeat-

Masker (Bedell et al.; 2000) identified transposable elements with a *de novo* library built by RepeatModeler (Abrusán et al.; 2009) and with a known library (Fugu) in RepBase (Bao et al.; 2015) using RMBlast.

Gene prediction and annotation

We combined *de novo*, RNA-based and homology-based methods to carry on protein-coding gene prediction. For the *de novo* and RNA-based gene prediction, Illumina RNA-seq and PacBio Iso-seq datasets were used to generate two hint files. Tophat (Kim et al.; 2013) aligned RNA-seq reads to the soft repeat-masked genome assembly. To obtain intron hints from Iso-seq, LSC (Au et al.; 2012) corrected sequencing errors in full-length transcripts with RNA-seq, GMAP (Wu and Watanabe; 2005) aligned the corrected transcripts to the genome, and gmap2hints.pl in the AUGUSTUS package (Stanke et al.; 2008) generated intron hints from the alignments. BRAKER (Brůna et al.; 2020) predicted protein-coding genes by incorporating the outputs of GeneMark-ET (Lomsadze et al.; 2014) and AUGUSTUS. GeneMark-ET predicts genes with unsupervised training, whereas AUGUSTUS predicts genes with supervised training based on intron and protein hints.

For the homology-based gene prediction, the assembly of *P. modestus* were aligned against the genes of 14 Actinopterygii genomes (Table S1) and vertebrata in orthoDB using TBLASTN (Camacho et al.; 2009) with an E-value cutoff of 1E-5. GenBlastA (She et al.; 2009) clustered matching sequences, and retained only best-matched regions which were used to predict gene models for a homology-based approach using Exonerate (Slater and Birney; 2005). Finally, the homology-based gene prediction were merged to the *ab initio* prediction only when there was no conflict. Then the merged genes were removed if their coding sequences (CDSs) contained premature stop codons or were not supported by hints. InterProScan (Jones et al.; 2014) annotated the predicted genes with various databases, including Hamap (Lima et al.; 2008), Pfam (Punta et al.; 2011), PIRSF (Nikolskaya et al.; 2007), PRINTS (Attwood et al.; 2000), ProDom (Bru et al.; 2005), PROSITE (Sigrist et al.; 2009), SUPERFAMILY (Madera et al.; 2004) and TIGRFAM (Haft et al.; 2012).

To predict non-coding genes, Infereal (Nawrocki and Eddy; 2013), RNAmmer (Lagesen et al.; 2007) and tRNAscan (Lowe and Eddy; 1997) were used.

Comparative genomics

Chromeister (Pérez-Wohlfeil et al.; 2019) performed all pairwise comparison with 17 Actinopterygii genomes to generate a synteny map. OrthoMCL (Li et al.; 2003) identified orthologous gene families among 15 Actinopterygii transcriptomes (Table S1). GO enrichment was performed using Fisher's exact test and false discovery rate correction to identify functionally enriched GO terms among gene families relative to the "genome background," as annotated by Pfam.

For phylogenetic analysis and divergence time estimation, MUSCLE (Edgar; 2004) aligned the amino acid sequences of single-copy gene families, trimAl (Capella-Gutiérrez et al.; 2009) filtered low alignment quality regions, RAxML (Stamatakis; 2014) constructed a phylogenetic tree with the PROTGAMMAJTT model (100 bootstrap replicates), and MEGA7 (Kumar et al.; 2016) calculated divergence time with the Jones-Taylor-Thornton model and the previously determined topology. Gene family expansion and contraction were analyzed by CAFE (Han et al.; 2013) with the identified orthologous gene families and the estimated phylogenetic information.

Table 1. Statistics of the genome assembly.

	Contigs	Scaffolds
# contigs ($\geq$ 0 bp)	3,839	1,419
# contigs ($\geq$ 10000 bp)	3,828	1,370
# contigs ($\geq$ 50000 bp)	2,784	581
Total length ($\geq$ 0 bp)	854,179,206	854,451,706
Total length ($\geq$ 10000 bp)	854,103,429	854,168,706
Total length ($\geq$ 50000 bp)	818,910,422	829,641,531
# contigs	3,839	1,419
Largest contig	5,687,114	44,673,496
Total length	854,179,206	854,451,706
GC (%)	40.64	40.64
N50	579,133	32,909,307
N75	227,794	28,196,589
L50	375	12
L75	953	19
# N's per 100 kbp	0.00	31.89

Results

Species identification

Comparison of Cytb sequences against the NCBI GenBank database (http://www.ncbi.nlm.nih.gov/) showed above 99% sequence identity to *P. modestus* (GenBank accession No. DQ901364.1), 89% to *P. argentilineatus* (AP019359.1) and 85% to *P. barbarus* (KF415633.1).

Chromosome-level genome assembly

We generated 39.6, 80.2, 52.9 and 33.3 Gbp (46, 94, 62, and 39 coverage) of Illumina, PacBio, 10X linked and Hi-C data, respectively, for genome sequencing (Table S2). The genome size was estimated at 729 Mbp using the 17-mer peak and distribution from cleaned Illumina data (Fig. S1). MiniMAP2 and MiniASM followed by polishing using RACON and Pilon generated 3,839 contigs (854 Mbp and N50 of 579 Kbp) using PacBio sequencing data. Tigmint, ARCS and LINKS generated 2,170 scaffolds (854 Mbp and N50 of 1.5 Mbp) using 10X linked data, and Dovetail HiRise finally generated 1,419 scaffolds including 23 pseudo-chromsomes (854 Mbp and N50 of 33 Mbp) using Hi-C data (Table 1). The pseudo-chormosomes were anchored by a Hi-C contact map (Fig. S2), and corresponded to the top 23 longest scaffolds of which the sum of lengths was close to the estimated genome size (742 Mb, Table S3). Interestingly, the number of pseudo-chromosomes is the same as that of chromosomes (Lee; 1986). Table 1 showed the length statistics of the genome assembly while Table S4 showed the genome completeness of 96.3% for contigs and scaffolds. Haplotigs and heterozygous overlaps of length 45 Mbp were purged, leaving 665 scaffolds (810 Mbp and N50 of 32.9 Mbp).

Genome annotation

Repetitive elements predicted by the three ways were merged to a total of 452 Mbp, which covered 44% of the genome: 11, 6, 5, 10 and 17% for DNA, LINE, simple repeat, tandem repeat and unknown, respectively (Table S5). We compared *P. modestus* with 16 Actinopterygii species for repeats (Table S7). As shown in Fig. 2, *P. modestus* had more simple and tandem repeats than the other Actinopterygii species.

For *ab initio* gene prediction, we generated 172 Gbp and 125 Mbp of RNA-seq and PacBio data, respectively, which yielded 366,298 and 131,807 hints for introns. BRAKER with GeneMark and AUGUSTUS predicted 132,821 genes. For homology-based gene prediction, we used 14 Actinopterygii species (Table S1). A pipeline of TBLASTN, GenBlastA and Exonerate predicted

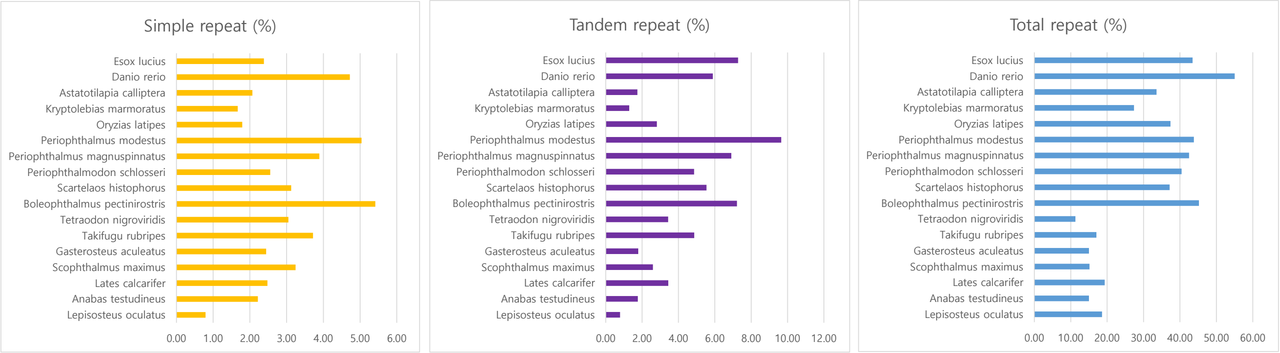

Figure 2. Percentage of the genome for simple, tandem and total repeats for 17 Actinopterygii species

22,721 genes. Merging the two outputs and filtering incomplete genes produced 30,505 genes and 34,916 transcripts (Table S6), of which 94% had homology to the 14 Actinopterygii transcriptomes. As a result of InterProScan annotation, 27,048 genes had 5,489 Pfam families, 25,995 genes had 5,121 InterPro domains, 17,310 genes had 2,277 GO terms, and 6,059 genes had 2,166 pathways.

Inferal predicted 5,071 non-coding genes such as lncRNA, miRNA, and misc RNA while tRNAscan predicted 4,510 tRNAs with 25 types (Table S11). RNAmmer predicted 1,950 rRNAs: 1836, 53 and 61 for 8s, 18s and 28s rRNA, respectively.

Synteny map

The 17 Actinopterygii genomes (Table S1) were compared to identify a synteny map using Chromeister. Fig. S3 shows dot plots in the upper triangular matrix and distance scores in the lower triangular matrix. As expected, the pair of *P. modestus* and *P. magnuspinnatus* had the lowest score, meaning the closest pair. The second and third lowest score corresponded to the pair of *B. pectinirostris* with *P. magnuspinnatus* and *P. modestus*, respectively. Note that the scores of *D. rerio* and *L. oculatus* with the others were greater than 0.99 because of the evolutionary distances.

Orthologous gene family

The 15 Actinopterygii whole-genome gene datasets (Table S1) were compared to identify orthologous gene families using orthoMCL. Among 59,448 gene families, 7,358 were common in all genomes, while 2265, 707, 792, 6461 2737 2070, 1082, 1059, 1576, 1751, 3326, 7326, 3389, 1901 and 1686 were only in *A. calliptera*, *A. testudineus*, *B. pectinirostris*, *D. rerio*, *E. lucius*, *G. aculeatus*, *K. marmoratus*, *L. calcarifer*, *L. oculatus*, *O. latipes*, *P. magnuspinnatus*, *P. modestus*, *S. maximus*, *T. nigroviridis* and *T. rubripes* respectively. As shown in Fig. 3, *P. modestus* had more families than the others and the number of common families in 13 or more species were dominant. The unique gene families of *P. modestus* enriched in negative regulation of RNA metabolic and biosynthetic process, nucleic acid-templated, transcription DNA-templated, nucleobase-containing, biosynthetic process, and celular macromolecule (Table S8).

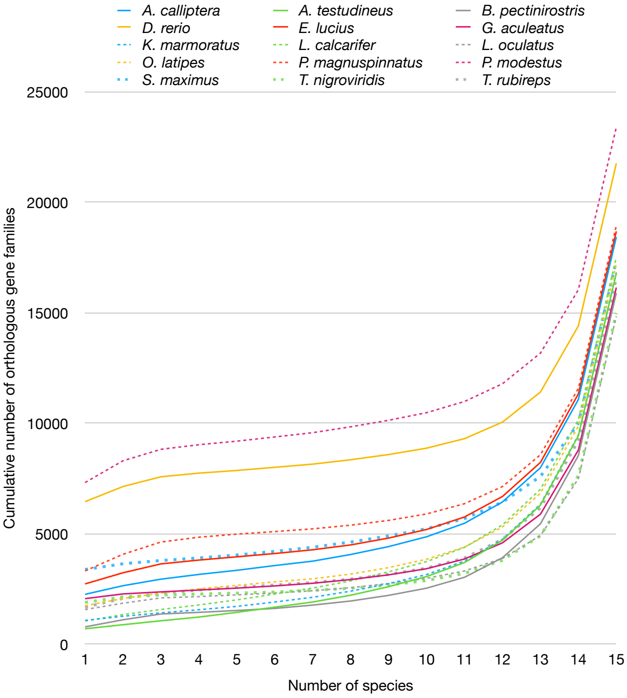

Figure 3. Cumulative number of orthologous gene families per the number of species w.r.t a specified species.

Phylogenetic relationships and divergence time

All genomes had 281 single-copy orthologous gene families which were used to construct a phylogenetic tree and estimate divergence time. The TimeTree database (Hedges et al.; 2006) was used to take calibration times between *L. calcarifer*–*S. maximus*, *K. marmoratus*–*O. latipes* and *T. rubireps*–*T. nigroviridis* divergence as 70–94, 76–114 and 42–59 MYA. As shown in Fig. 4, the infraclass Teleostei was separated at ~320MYA, consistent to the previous study (Betancur-R et al.; 2017), the order Cypriniformes at ~287MYA, the order Esociformes at ~224MYA, and the order Gobiiformes at ~141MYA. *P. modestus* clustered with the other species in the order Gobiiformes, and diverged from *P. magnuspinnatus* and *B. pectinirostris* during the late and mid Cenozoic era (15 and 25 MYA), respectively.

Gene family expansion and contraction

Orthologous gene families among the 15 Actinopterygii genomes were used for analyzing gene family expansion and contraction. The number of expanded and contracted gene families of *P. modestus* with its common ancestor were 411 and 225 while those of *P. magnuspinnatus*, the closest genome, were 257 and 442, respectively (Fig. 4). The expanded gene families of *P. modestus* were enriched in base-excision repair, transmembrane receptor protein tyrosine kinase signaling pathway, and enzyme linked receptor protein signaling pathway (Table S9) while the contracted gene families of *P. modestus* were in FMN binding, ion binding, and reactive oxygen species metabolic

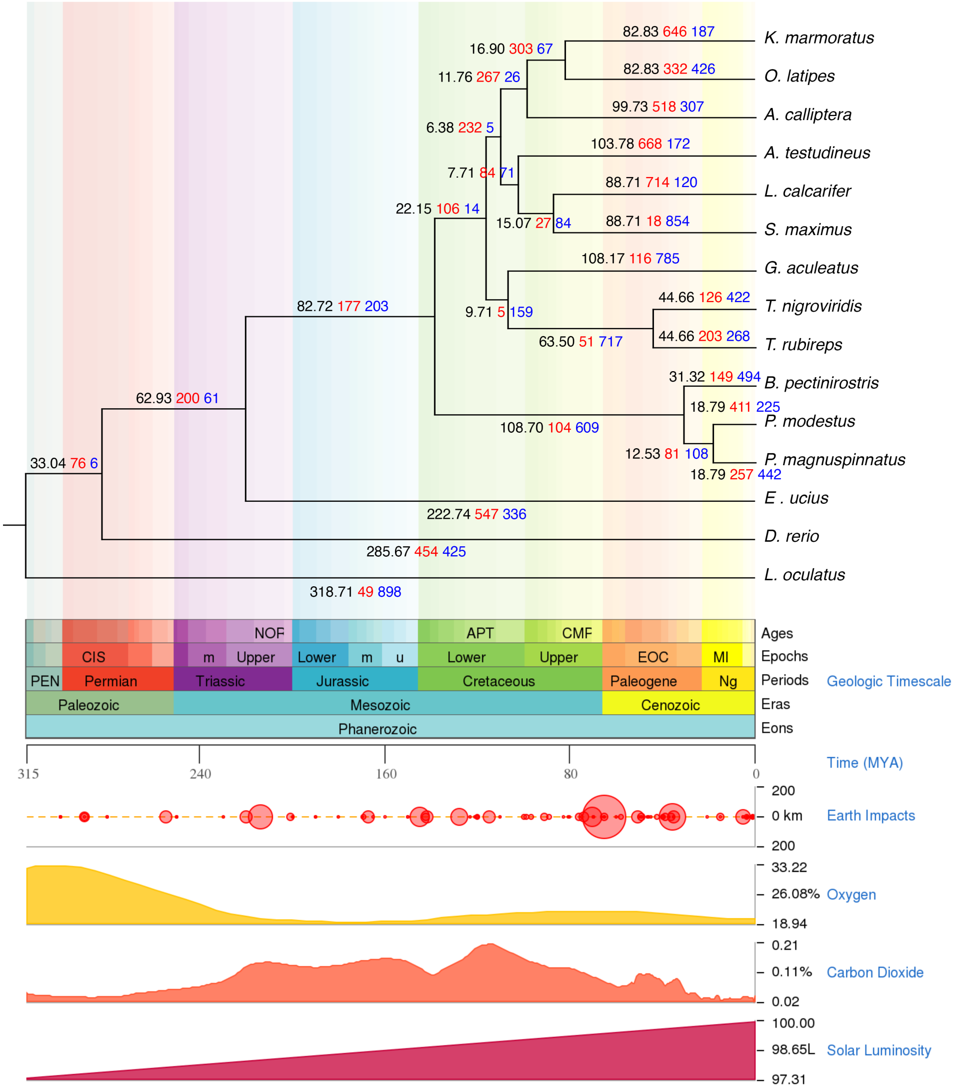

Figure 4. Time tree was constructed by MEGA7 with 281 single-copy orthologous gene families among 15 Actinopterygii where the first (black) numbers represent divergence time in million of years; the second (red) and third (blue) numbers represent the number of expanded and contracted, respectively, gene families identified by CAFE; the geologic timescale, earth impacts, oxgen, cabor dioxide and solar luminosity were generated on the TimeTree database.

process (Table S10). Fig. S4 shows word cloud for GO term description enriched in unique, expanded and contracted gene families of *P. modestus*.

Conclusions

We presented a chromosome-level high-quality genome assembly of *P. modestus* with N50 length of 33 Mbp using Illumina, PacBio, 10X, Hi-C, RNA and Isoform sequencing respectively. The completeness of the genome was confirmed by the BUSCO score of 96.3%. The top 23 longest scaffolds were above 20 Mbp in size and close to the estimated genome size of 728 Mbp. *P. modestus* had various repetitive elements in 43.8% of the genome and more repetitive elements than the 16 Actinopterygii genomes. We predicted 34,871 protein coding and 7,865 non-coding genes, and 93% of the protein coding genes had homology to the 14 Actinopterygii transcriptomes. This dataset will provide a valuable resource for further studies on sea-land transition, bimodal respiration, nitrogen excretion, osmoregulation, thermoregulation, vision and mechanoreception.

Availability of source code and requirements

Table S12 shows the software versions, settings and parameters.

Availability of supporting data and materials

All raw sequencing reads have been deposited in the NCBI SRA (Table S2) under BioProject No. PRJNA660579. The assembled genome was submitted to NCBI Assembly. Gene annotation and transcript sequences were provides as supplementary files. JBrowse (Buels et al.; 2016) was set up on `http://magic.re.kr/gbrowser/jb/mabik/?data=shuttles_hoppfish`.

Additional files

Supplementary figures:

Fig. S1. Genome size estimation by 17-mer distribution.
Fig. S2. Hi-C contact map.
Fig. S3. Synteny map of 17 Actinopterygii genomes.
Fig. S4. Word cloud for GO term description.

Supplementary tables:

Table S1. Taxonomy and statistics of 17 Actinopterygii species.
Table S2. Statistics of sequencing data.
Table S3. Top 23 longest scaffolds.
Table S4. BUSCO assessment of genome assembly and gene prediction with metazoa.
Table S5. Statistics of repetitive elements.
Table S6. Statistics of predicted protein-coding genes.
Table S7. Repeat analysis for the 17 Actinopterygii genome.
Table S8. Top 40 GO terms enriched in unique gene families of *P. modestus*.
Table S9. Top 40 GO terms enriched in expanded gene families of *P. modestus*.
Table S10. Top 40 GO terms enriched in contracted gene families of *P. modestus*.
Table S11. Statistics of predicted non-coding genes.
Table S12. A list of software and parameters used for genome analyses.

Declarations

List of abbreviations

Cytb: cytochrome b; PCR: polymerase chain reaction; RNA-seq: RNA sequencing; Iso-seq: Isoform sequencing; Kbp: killo base pair; Mbp: mega base pair; Gbp: giga base pair; GO: gene ontology; MYA: million years ago.

Competing Interests

The authors declare no competing interests.

Funding

This study was financially supported by the National Marine Biodiversity Institute of Korea Research Program (2021M00600).

Author's Contributions

J.H.C and Y.Y. conceived concept, H.Y.S., S.J., and S.H.J collected and classified the sample, J.H.C. and Y.Y. designed the experiments, J.Y.Y., S.H.B, Y.Y. and J.H.C. analyzed the genomic data, S.H.B. and Y.Y deposited the data into NCBI, H.Y.S., Y.Y. and J.H.C. wrote the paper. All authors reviewed the manuscript.

References

Abrusán, G., Grundmann, N., DeMester, L. and Makalowski, W. (2009). TEclass—a tool for automated classification of unknown eukaryotic transposable elements, *Bioinformatics* **25**(10): 1329–1330.

Attwood, T. K., Croning, M. D. R., Flower, D. R., Lewis, A. P., Mabey, J. E., Scordis, P., Selley, J. N. and Wright, W. (2000). PRINTS-S: the database formerly known as PRINTS, *Nucleic Acids Research* **28**(1): 225–227.

Au, K. F., Underwood, J. G., Lee, L. and Wong, W. H. (2012). Improving pacbio long read accuracy by short read alignment, *PLOS ONE* **7**(10): 1–8.

Bao, W., Kojima, K. K. and Kohany, O. (2015). Repbase Update, a database of repetitive elements in eukaryotic genomes, *Mobile DNA* **6**: 11.

Bedell, J. A., Korf, I. and Gish, W. (2000). MaskerAid : a performance enhancement to RepeatMasker , *Bioinformatics* **16**(11): 1040–1041.

Benson, G. (1999). Tandem repeats finder: a program to analyze DNA sequences, *Nucleic Acids Research* **27**(2): 573–580.

Betancur-R, R., Wiley, E. O., Arratia, G., Acero, A., Bailly, N., Miya, M., Lecointre, G. and Ortí, G. (2017). Phylogenetic classification of bony fishes, *BMC Evolutionary Biology* **17**: 162.

Bolger, A. M., Lohse, M. and Usadel, B. (2014). Trimmomatic: a flexible trimmer for Illumina sequence data, *Bioinformatics* **30**(15): 2114–2120.

Bru, C., Courcelle, E., Carrère, S., Beausse, Y., Dalmar, S. and Kahn, D. (2005). The ProDom database of protein domain families: more emphasis on 3D, *Nucleic Acids Research* **33**(suppl1): D212–D215.

Brůna, T., Hoff, K. J., Lomsadze, A., Stanke, M. and Borodovsky, M. (2020). Braker2: Automatic eukaryotic genome annotation with genemark-ep+ and augustus supported by a protein database, *bioRxiv* .

Buels, R., Yao, E., Diesh, C. M., Hayes, R. D., Munoz-Torres, M., Helt, G., Goodstein, D. M., Elsik, C. G., Lewis, S. E., Stein, L. and Holmes, I. H. (2016). Jbrowse: a dynamic web platform for genome visualization and analysis, *Genome Biology* **17**: 66.

Camacho, C., Coulouris, G., Avagyan, V., Ma, N., Papadopoulos, J., Bealer, K. and Madden, T. L. (2009). BLAST+: architecture and applications, *BMC Bioinformatics* **10**: 421.

Cantor, T. E. (1842). General features of chusan, with remarks on the flora and fauna of that island, *Annuals and Magazine of Natural History* **9**(58,59,60): 265–278, 361–370, 481–493.

Capella-Gutiérrez, S., Silla-Martínez, J. M. and Gabaldón, T. (2009). trimAl: a tool for automated alignment trimming in large-scale phylogenetic analyses, *Bioinformatics* **25**(15): 1972–1973.

Chen, W., Hong, W., Chen, S., Wang, Q. and Zhang, Q. (2015). Population genetic structure and demographic history of the mudskipper boleophthalmus pectinirostris on the northwestern pacific coast, *Environmental Biology of Fishes* **98**(3): 845–856.

Edgar, R. C. (2004). MUSCLE: multiple sequence alignment with high accuracy and high throughput, *Nucleic Acids Research* **32**(5): 1792–1797.

Guan, D., McCarthy, S. A., Wood, J., Howe, K., Wang, Y. and Durbin, R. (2020). Identifying and removing haplotypic duplication in primary genome assemblies, *Bioinformatics* **36**(9): 2896–2898.

Gurevich, A., Saveliev, V., Vyahhi, N. and Tesler, G. (2013). QUAST: quality assessment tool for genome assemblies, *Bioinformatics* **29**(8): 1072–1075.

Haft, D. H., Selengut, J. D., Richter, R. A., Harkins, D., Basu, M. K. and Beck, E. (2012). TIGRFAMs and Genome Properties in 2013, *Nucleic Acids Research* **41**(D1): D387–D395.

Han, M. V., Thomas, G. W., Lugo-Martinez, J. and Hahn, M. W. (2013). Estimating Gene Gain and Loss Rates in the Presence of Error in Genome Assembly and Annotation Using CAFE 3, *Molecular Biology and Evolution* **30**(8): 1987–1997.

Hedges, S. B., Dudley, J. and Kumar, S. (2006). TimeTree: a public knowledge-base of divergence times among organisms, *Bioinformatics* **22**(23): 2971–2972.

Jackman, S. D., Coombe, L., Chu, J., Warren, R. L., Vandervalk, B. P., Yeo, S., Xue, Z., Mohamadi, H., Bohlmann, J., Jones, S. J. M. and Birol, I. (2018). Tigmint: correcting assembly errors using linked reads from large molecules, *BMC Bioinform.* **19**(1): 393:1–393:10.

Jones, P., Binns, D., Chang, H.-Y., Fraser, M., Li, W., McAnulla, C., McWilliam, H., Maslen, J., Mitchell, A., Nuka, G., Pesseat,

S., Quinn, A. F., Sangrador-Vegas, A., Scheremetjew, M., Yong, S.-Y., Lopez, R. and Hunter, S. (2014). InterProScan 5: genome-scale protein function classification, *Bioinformatics* **30**(9): 1236–1240.

Kim, D., Pertea, G., Trapnell, C., Pimentel, H., Kelley, R. and Salzberg, S. L. (2013). TopHat2: accurate alignment of transcriptomes in the presence of insertions, deletions and gene fusions, *Genome Biology* **14**: R36.

Kumar, S., Stecher, G. and Tamura, K. (2016). MEGA7: Molecular Evolutionary Genetics Analysis Version 7.0 for Bigger Datasets, *Molecular Biology and Evolution* **33**(7): 1870–1874.

Lagesen, K., Hallin, P., Rødland, E. A., Stærfeldt, H.-H., Rognes, T. and Ussery, D. W. (2007). RNAmmer: consistent and rapid annotation of ribosomal RNA genes, *Nucleic Acids Research* **35**(9): 3100–3108.

Lee, G. (1986). Karyotypes of the family gobiidae fishes in korea (i), *Korea J. Limnol.* **19**: 49–58.

Li, H. (2016). Minimap and miniasm: fast mapping and de novo assembly for noisy long sequences, *Bioinformatics* **32**(14): 2103–2110.

Li, H. (2018). Minimap2: pairwise alignment for nucleotide sequences, *Bioinformatics* **34**(18): 3094–3100.

Li, H. and Durbin, R. (2009). Fast and accurate short read alignment with Burrows–Wheeler transform, *Bioinformatics* **25**(14): 1754–1760.

Li, L., Stoeckert, C. J. and Roos, D. S. (2003). Orthomcl: Identification of ortholog groups for eukaryotic genomes, *Genome Research* **13**(9): 2178–2189.

Lima, T., Auchincloss, A. H., Coudert, E., Keller, G., Michoud, K., Rivoire, C., Bulliard, V., de Castro, E., Lachaize, C., Baratin, D., Phan, I., Bougueleret, L. and Bairoch, A. (2008). HAMAP: a database of completely sequenced microbial proteome sets and manually curated microbial protein families in UniProtKB/Swiss-Prot, *Nucleic Acids Research* **37**(suppl1): D471–D478.

Lomsadze, A., Burns, P. D. and Borodovsky, M. (2014). Integration of mapped RNA-Seq reads into automatic training of eukaryotic gene finding algorithm, *Nucleic Acids Research* **42**(15): e119–e119.

Lowe, T. M. and Eddy, S. R. (1997). tRNAscan-SE: A Program for Improved Detection of Transfer RNA Genes in Genomic Sequence, *Nucleic Acids Research* **25**(5): 955–964.

Madera, M., Vogel, C., Kummerfeld, S. K., Chothia, C. and Gough, J. (2004). The SUPERFAMILY database in 2004: additions and improvements, *Nucleic Acids Research* **32**(suppl1): D235–D239.

Marçais, G. and Kingsford, C. (2011). A fast, lock-free approach for efficient parallel counting of occurrences of k-mers, *Bioinformatics* **27**(6): 764–770.

Nawrocki, E. P. and Eddy, S. R. (2013). Infernal 1.1: 100-fold faster RNA homology searches, *Bioinformatics* **29**(22): 2933–2935.

Nelson, J. S., Grande, T. C. and Wilson, M. V. H. (2016). *Fishes of the World*, John Wiley & Sons, Ltd.

Nikolskaya, A. N., Arighi, C. N., Huang, H., Barker, W. C. and Wu, C. H. (2007). Pirsf family classification system for protein functional and evolutionary analysis, *Evolutionary bioinformatics online* **2**: 197–209.

Parenti, L. R. and Jaafar, Z. (2017). *The Natural Distribution of Mudskippers*, CRC Press, chapter 2.

Punta, M., Coggill, P. C., Eberhardt, R. Y., Mistry, J., Tate, J., Boursnell, C., Pang, N., Forslund, K., Ceric, G., Clements, J., Heger, A., Holm, L., Sonnhammer, E. L. L., Eddy, S. R., Bateman, A. and Finn, R. D. (2011). The Pfam protein families database, *Nucleic Acids Research* **40**(D1): D290–D301.

Putnam, N. H., O'Connell, B. L., Stites, J. C., Rice, B. J., Blanchette, M., Calef, R., Troll, C. J., Fields, A., Hartley, P. D., Sugnet, C. W., Haussler, D., Rokhsar, D. S. and Green, R. E. (2016). Chromosome-scale shotgun assembly using an in vitro method for long-range linkage, *Genome Research* **26**(3): 342–350.

Pérez-Wohlfeil, E., del Pino, S. D. and Trelles, O. (2019). Ultra-fast genome comparison for large-scale genomic experiments, *Scientific Reports* **9**: 10274.

She, R., Chu, J. S.-C., Wang, K., Pei, J. and Chen, N. (2009). genblasta: Enabling blast to identify homologous gene sequences, *Genome Research* **19**(1): 143–149.

Sigrist, C. J. A., Cerutti, L., de Castro, E., Langendijk-Genevaux, P. S., Bulliard, V., Bairoch, A. and Hulo, N. (2009). PROSITE, a protein domain database for functional characterization and annotation, *Nucleic Acids Research* **38**(suppl1): D161–D166.

Simão, F. A., Waterhouse, R. M., Ioannidis, P., Kriventseva, E. V. and Zdobnov, E. M. (2015). BUSCO: assessing genome assembly and annotation completeness with single-copy orthologs, *Bioinformatics* **31**(19): 3210–3212.

Slater, G. S. C. and Birney, E. (2005). Automated generation of heuristics for biological sequence comparison, *BMC Bioinformatics* **6**: 31.

Stamatakis, A. (2014). RAxML version 8: a tool for phylogenetic analysis and post-analysis of large phylogenies, *Bioinformatics* **30**(9): 1312–1313.

Stanke, M., Diekhans, M., Baertsch, R. and Haussler, D. (2008). Using native and syntenically mapped cDNA alignments to improve de novo gene finding, *Bioinformatics* **24**(5): 637–644.

Thacker, C. E. and Roje, D. M. (2011). Phylogeny of gobiidae and identification of gobiid lineages, *Systematics and Biodiversity* **9**(4): 329–347.

Vaser, R., Sović, I., Nagarajan, N. and Šikić, M. (2017). Fast and accurate de novo genome assembly from long uncorrected reads, *Genome Research* **27**(5): 737–746.

Vurture, G. W., Sedlazeck, F. J., Nattestad, M., Underwood, C. J., Fang, H., Gurtowski, J. and Schatz, M. C. (2017). GenomeScope: fast reference-free genome profiling from short reads, *Bioinformatics* **33**(14): 2202–2204.

Walker, B. J., Abeel, T., Shea, T., Priest, M., Abouelliel, A., Sakthikumar, S., Cuomo, C. A., Zeng, Q., Wortman, J., Young, S. K. and Earl, A. M. (2014). Pilon: An integrated tool for comprehensive microbial variant detection and genome assembly improvement, *PLOS ONE* **9**(11): 1–14.

Warren, R. L., Yang, C., Vandervalk, B. P., Behsaz, B., Lagman, A., Jones, S. J. M. and Birol, I. (2015). LINKS: Scalable, alignment-free scaffolding of draft genomes with long reads, *GigaScience* **4**(1). s13742-015-0076-3.

Wicaksono, A., Hidayat, S., Retnoaji, B. and Alam, P. (2020). The water-hopping kinematics of the tree-climbing fish, *Periophthalmus variabilis*, *Zoology* **139**: 125750.

Wu, T. D. and Watanabe, C. K. (2005). GMAP: a genomic mapping and alignment program for mRNA and EST sequences, *Bioinformatics* **21**(9): 1859–1875.

Yeo, S., Coombe, L., Warren, R. L., Chu, J. and Birol, I. (2017). ARCS: scaffolding genome drafts with linked reads, *Bioinformatics* **34**(5): 725–731.

You, X., Bian, C., Zan, Q., Xu, X., Liu, X., Chen, J., Wang, J., Qiu, Y., Li, W., Zhang, X., Sun, Y., Chen, S., Hong, W., Li, Y., Cheng, S., Fan, G., Shi, C., Liang, J., Tom Tang, Y., Yang, C., Ruan, Z., Bai, J., Peng, C., Mu, Q., Lu, Jun andFan, M., Yang, S., Huang, Z., Jiang, X., Fang, X., Zhang, G., Zhang, Y., Polgar, G., Yu, H., Li, J., Liu, Z., Zhang, G., Ravi, V., Coon, S. L., Wang, J., Yang, H., Venkatesh, B., Wang, J. and Shi, Q. (2014). Mudskipper genomes provide insights into the terrestrial adaptation of amphibious fishes, *Nature Communications* **5**: 5594.

Supplementary tables

Click here to access/download
Supplementary Material
supplementary_tables.pdf

Supplementary figures

Click here to access/download
Supplementary Material
supplementary_figures.pdf

Click here to access/download;Personal Cover;review-response.pdf

We very much appreciate the reviewer for precise and valuable comments.

Reviewer #2: The most responses from the authors present an agreement with my previous opinions, and I appreciate about this. However, the authors also raise a question about gene model annotation, which may be understood differently from my thought. I understand that Ab Initio gene prediction is an intrinsic method based on gene content and signal detection, thus not include the RNA-based annotation. The consideration of Ab Initio gene prediction from the authors may include the de novo and RNA prediction. This is the controversial point. According to my opinion, the opening sentence should be rephrased as "We combined the method of de novo, RNA-based and homology-based to carry on the gene model prediction."

The manuscript in section Methods was revised as suggested, "We combined de novo, RNA-based and homology-based methods to carry on protein-coding gene prediction. For the de novo and RNA-based gene prediction,"
