# Supplementary material for: Chromosome-level genome assembly of the shuttles hoppfish, Periophthalmus modestus
Source: Gigascience. 2022 Jan 12;11:giab089. doi: 10.1093/gigascience/giab089 (PMC8756193; doi:10.1093/gigascience/giab089)
Supplement: giab089_Supplemental_Figures_and_Tables [file giab089_supplemental_figures_and_tables.zip › supplementary_figures.pdf]

Chromosome-level genome assembly of the shuttles hoppfish, *Periophthalmus modestus*

Youngik Yang, Ji Yong Yoo, Sang Ho Baek, Ha Yeun Song,
Seonmi Jo, Seung-Hyun Jung & Jeong-Hyeon Choi

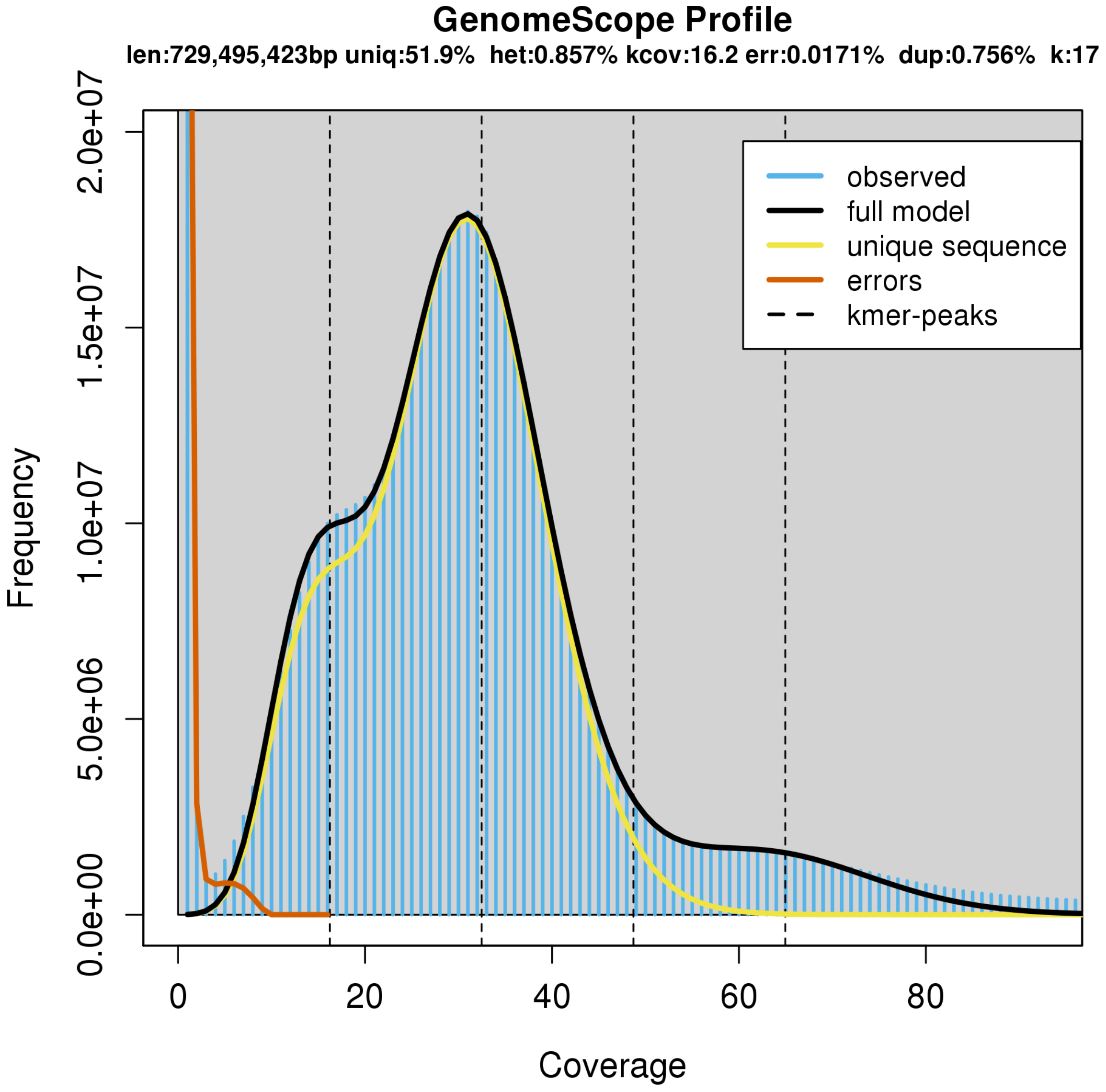

Figure S1: Genome size estimation by 17-mer distribution.

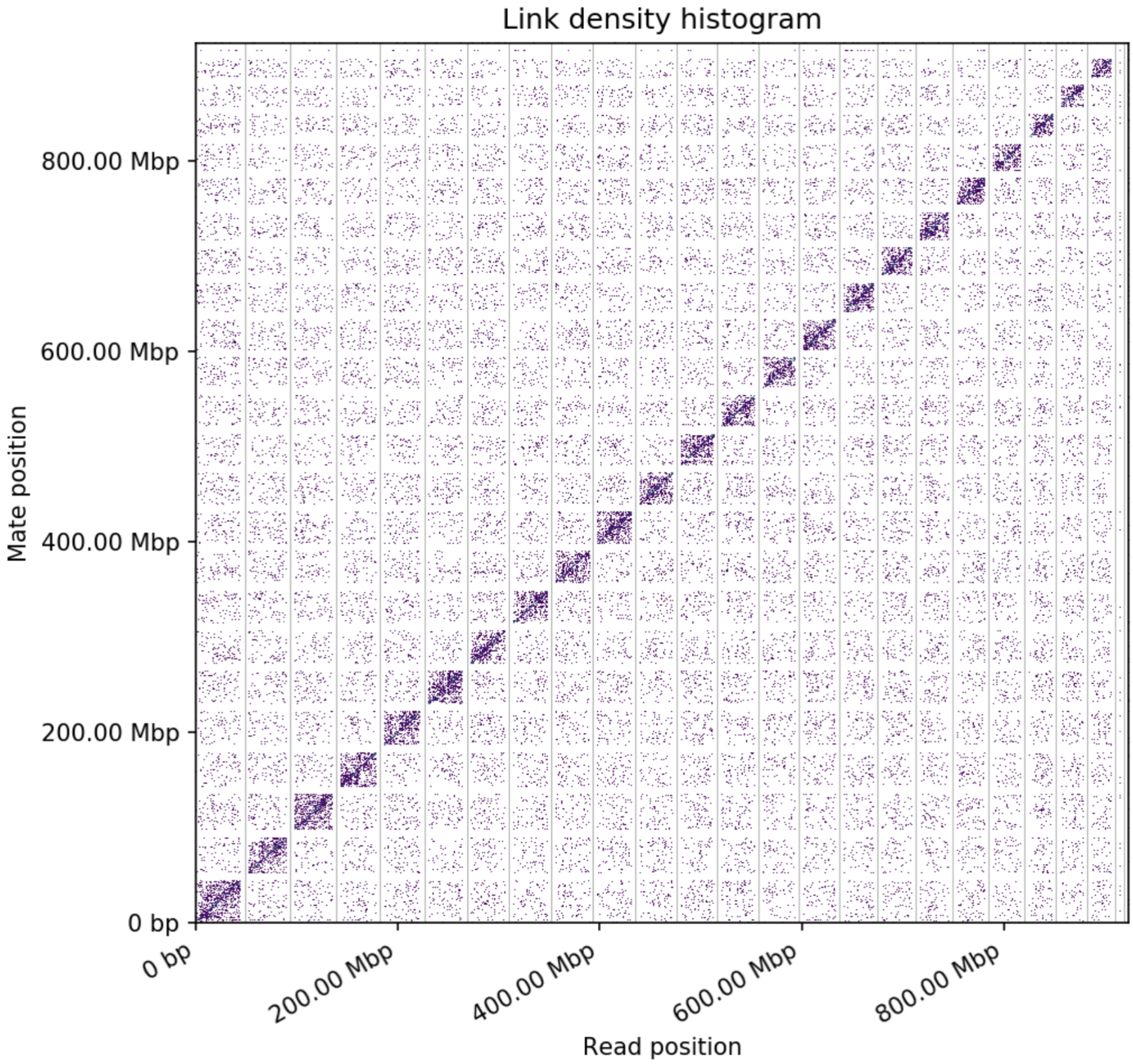

Figure S2: Hi-C contact map. The x and y axes represent the mapping positions of the first and second read in the read pair respectively, grouped into bins. The color of each square corresponds to the number of read pairs within that bin. White vertical and black horizontal lines were added to show the borders between scaffolds. Scaffolds less than 1 Mb were excluded.

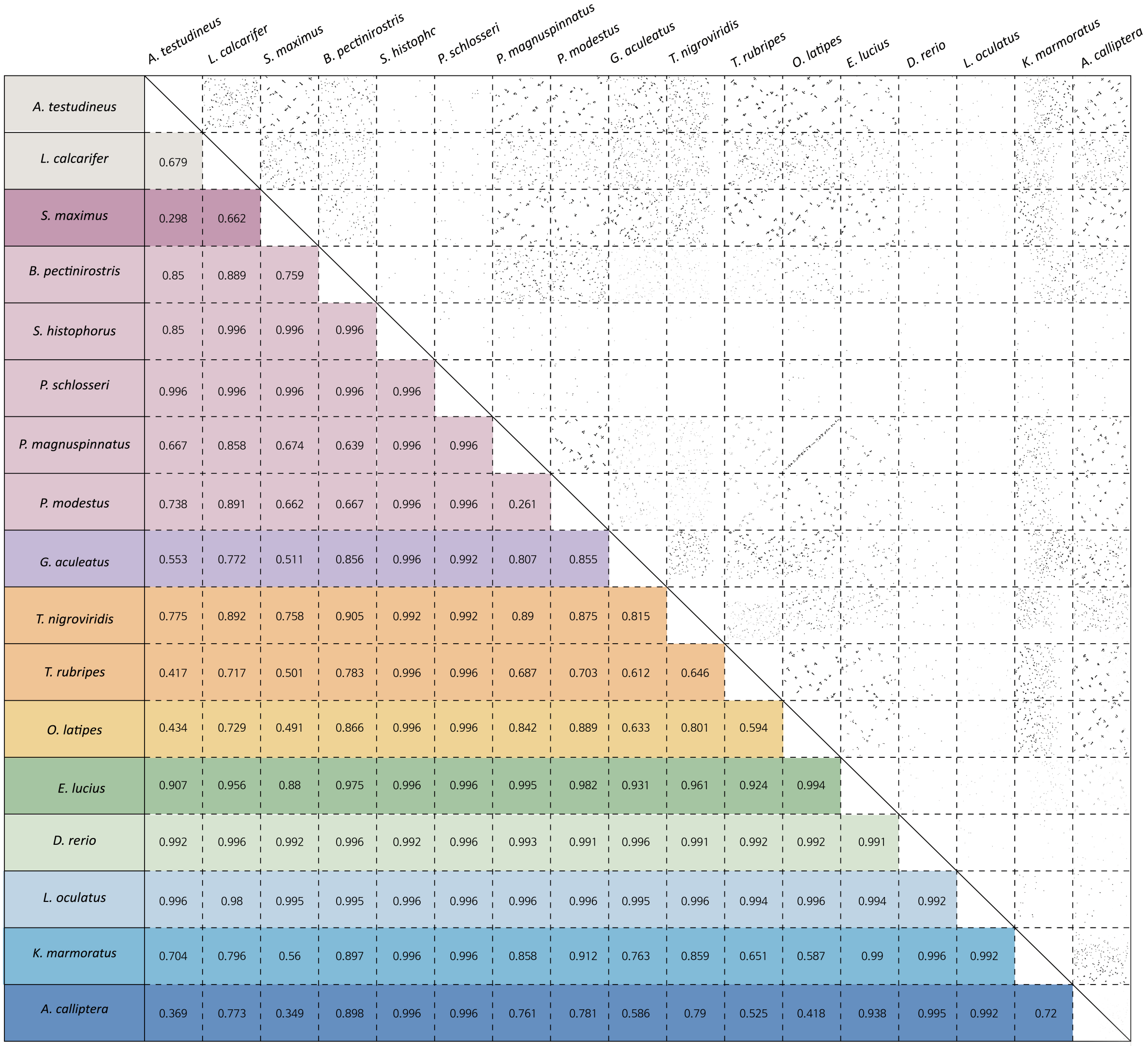

Figure S3: Synteny map of 17 Actinopterygii genomes where the dots and numbers in the upper and lower triangular matrix represent similar regions and distance score, respectively, between two genomes, and the colors represent their order.

(a)

(b)

(c)

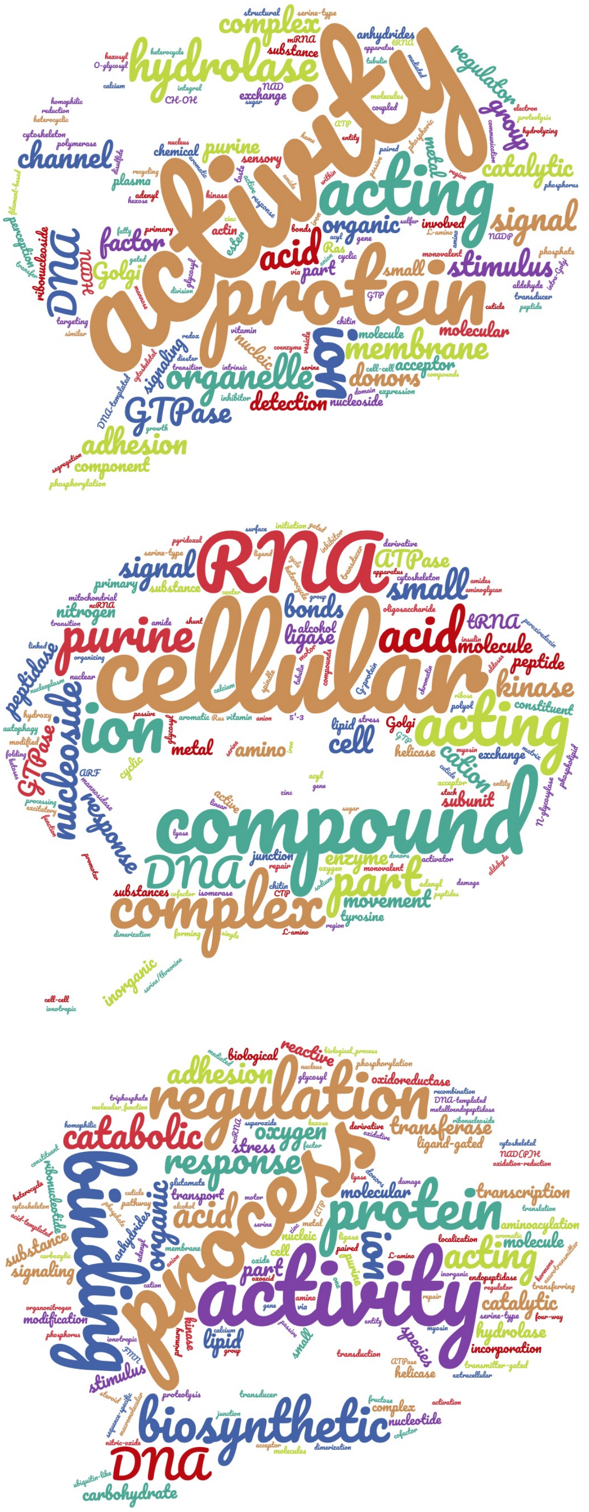

Figure S4: Word cloud for GO term description enriched in (a) unique, (b) expanded and (c) contracted gene families of *P. modestus*.
