# Supplementary material for: Chromosome-level genome assembly of the shuttles hoppfish, Periophthalmus modestus
Source: Gigascience. 2022 Jan 12;11:giab089. doi: 10.1093/gigascience/giab089 (PMC8756193; doi:10.1093/gigascience/giab089)
Supplement: giab089_Supplemental_Figures_and_Tables [file giab089_supplemental_figures_and_tables.zip › supplementary_tables.pdf]

Chromosome-level genome assembly of the shuttles hoppfish, *Periophthalmus modestus*

Youngik Yang, Ji Yong Yoo, Sang Ho Baek, Ha Yeun Song,
Seonmi Jo, Seung-Hyun Jung & Jeong-Hyeon Choi

Table S1: Taxonomy and statistics of 17 Actinopterygii species used for analysis
where superscript 1, 2, 3 and 4 following scientific names represent species used for repeat analysis, homology-based gene prediction, orthologous gene family analysis, and evolutionary tree, respectively.

Scietific name	Common name	Taxonomy		
		infraclass	order	family
Lepisosteus oculatus	spotted gar	Holostei	Semionotiformes	Lepisosteidae
Anabas testudineus	climbing perch	Teleostei	Anabantiformes	Anabantidae
Lates calcarifer	barramundi perch			Centropomidae
Scophthalmus maximus	turbot		Pleuronectiformes	Scophthalmidae
Gasterosteus aculeatus	three-spined stickleback		Perciformes	Gasterosteidae
Takifugu rubripes	torafugu		Tetraodontiformes	Tetraodontidae
Tetraodon nigroviridis	spotted green pufferfish			
Boleophthalmus pectinirostris	great blue-spotted hoppfish		Gobiiformes	Gobiidae
Scartelaos histophorus	walking goby			
Periophthalmodon schlosseri	giant hoppfish			
Periophthalmus magnuspinnatus				
Periophthalmus modestus	shuttles hoppfish			
Oryzias latipes	Japanese medaka		Beloniformes	Adrianichthyidae
Kryptolebias marmoratus	mangrove rivulus		Cyprinodontiformes	Rivulidae
Astatotilapia calliptera	eastern happy		Cichliformes	Cichlidae
Danio rerio	zebrafish		Cypriniformes	Cyprinidae
Esox lucius	northern pike		Esociformes	Esocidae

Scietific name	Genome			Transcript	
	Accession	Number	Length	Number	Length
Lepisosteus oculatus[1,2,3,4]	GCF_000242695.1	1,926	945,878,036	22,460	13,405,485
Anabas testudineus[1,2,3,4]	GCF_900324465.1	70	569,722,067	40,344	28,570,267
Lates calcarifer[1,2,3,4]	GCF_001640805.1	3,808	668,481,366	45,186	31,938,764
Scophthalmus maximus[1,2,3,4]	GCA_003186165.1	22	524,979,463	24,929	14,009,638
Gasterosteus aculeatus[1,2,3,4]	GCA_006229165.1	10,242	467,452,432	27,249	13,490,636
Takifugu rubripes[1,2,3,4]	GCF_901000725.2	128	384,126,662	27,904	14,720,522
Tetraodon nigroviridis[1,2,3,4]	GCA_000180735.1	25,773	342,403,326	23,074	12,616,183
Boleophthalmus pectinirostris[1,2,3,4]	GCF_000788275.1	16,620	955,752,150	25,128	14,772,731
Scartelaos histophorus[1]	GCA_000787155.1	156,044	695,008,792		
Periophthalmodon schlosseri[1]	GCA_000787095.1	46,662	679,761,122		
Periophthalmus magnuspinnatus[1,2,3]	GCA_009829125.1	124	752,621,857	30,051	13,008,771
Periophthalmus modestus[1,2]		1,419	854,451,706	34,871	18,654,191
Oryzias latipes[1,2,3,4]	GCF_002234675.1	25	734,057,086	36,143	21,724,021
Kryptolebias marmoratus[1,2,3]	GCF_001649575.1	3,073	680,366,784	42,343	31,938,764
Astatotilapia calliptera[1,2,3]	GCF_900246225.1	249	880,445,564	40,006	23,142,894
Danio rerio[1,2,3,4]	GCF_000002035.6	1,923	1,679,203,469	46,913	24,593,863
Esox lucius[1,2,3,4]	GCF_004634155.1	811	940,906,975	41,204	24,441,917

Table S2: Statistics of sequencing data.

Platform	Reads	Total bases (bp)	Average length (bp)	N50 length (bp)	SRA accession
Ilumina	262,479,000	39,634,329,000	151	151	SRR12825117
PacBio	8,637,678	80,233,962,214	9,288	17,877	SRR12825100-7<br>SRR12825114-6
10X	352,521,790	52,878,268,500			SRR15068217
Hi-C	220,455,494	33,288,779,594			SRR15068216
RNA-seq	623,638,468	172,746,559,714	101	101	SRR12825114-20
Iso-seq	48,319	125,105,012	2,589	2,898	SRR13214314

Table S3: Top 23 longest scaffolds.

No.	Length (bp)	% of assembly
1	44,673,496	5.23
2	37,769,314	4.42
3	37,702,436	4.41
4	36,347,356	4.25
5	35,966,534	4.21
6	34,678,682	4.06
7	34,462,777	4.03
8	34,397,552	4.03
9	34,123,802	3.99
10	34,086,128	3.99
11	33,827,149	3.96
12	32,909,307	3.85
13	32,822,805	3.84
14	32,745,397	3.83
15	32,669,072	3.82
16	30,362,753	3.55
17	29,743,397	3.48
18	29,460,102	3.45
19	28,196,589	3.30
20	27,745,997	3.25
21	24,417,265	2.86
22	23,011,381	2.69
23	20,371,859	2.38
Total	742,491,150	86.90

Table S4: BUSCO assessment of genome assembly and gene prediction with metazoa.

C:97.0%[S:93.3%,D:3.7%],F:0.8%,M:2.2%,n:954
C:96.6%[S:93.9%,D:2.7%],F:1.0%,M:2.4%,n:954
C:92.8%[S:82.6%,D:10.2%],F:4.2%,M:3.0%,n:954

Table S5: Statistics of repetitive elements.

Class	Number	Total length(bp)	% of genome
ARTEFACT	1	53	0.0
DNA	430,612	89,454,735	10.5
LINE	258,657	54,828,280	6.4
LTR	46,816	12,343,074	1.4
Low complexity	111,096	8,785,913	1.0
Other	6	11,549	0.0
Retroposon	1	183	0.0
SINE	40,319	6,372,179	0.7
Satellite	22,262	4,807,537	0.6
Simple repeat	717,579	43,054,166	5.0
Tandem repeat	672,936	82,463,320	9.7
Unknown	793,859	145,959,105	17.1
Unspecified	23,048	4,108,047	0.5
Overlapping total	3,117,192	452,188,141	52.9
Non-overlapping total		374,339,026	43.8

Table S6: Statistics of predicted protein-coding genes.

	Number of genes	Number of transcripts	Number of non-overlapping exons	Number of non-overlapping introns	Average number of exons per transcript	Number of alternatively spliced genes
Ab initio	27,681	32,092	271,121	240,512	10.9	3,351
Homology	22,721	22,721	176,970	154,799	7.9	0
	2,824	2,824	6834	4,030	2.4	0
Merged	30,505	34,916	277,953	244,542	10.2	3,351

	Number of single exon transcripts	Average gene length (bp)	Average transcript length (bp)		Average exon length (bp)	Average intron length (bp)
			w/ introns	w/o introns		
Ab initio	0	10,278.9	11,410.9	1,670.4	155.1	1,010.6
Homology	2,667	10,615.1	10,615.1	1,298.9	164.2	1,354.5
	1,632	4,452.0	4,452.0	869.2	357.2	2,502.5
Merged	1,632	9,739.5	10,848.1	1,605.6	160.0	1,034.6

Table S7: Repeat analysis for the 17 Actinopterygii genomes where the numbers represent the percentage of the genome

Scientific name	DNA	LINE	LTR	Low comple xity	Other	SINE	Satellite	Simple repeat	Tandem repeat	Unknown	Unspecified	Total
Lepisosteus oculatus	3.86	4.75	1.10	0.14	0.00	2.03	0.06	0.79	0.79	5.84	0.12	18.60
Anabas testudineus	4.91	3.68	0.86	0.34	0.00	0.16	0.09	2.22	1.76	3.05	0.28	14.99
Lates calcarifer	6.09	1.89	1.25	0.40	0.00	0.05	0.26	2.48	3.44	6.39	0.29	19.32
Scophthalmus maximus	5.50	1.90	0.68	0.37	0.00	0.17	0.11	3.25	2.60	3.39	0.41	15.18
Gasterosteus aculeatus	3.69	2.71	3.50	0.30	0.00	0.11	0.06	2.44	1.78	2.37	0.29	15.02
Takifugu rubripes	3.55	4.90	2.10	0.32	0.00	0.03	0.29	3.72	4.87	1.81	0.46	17.06
Tetraodon nigroviridis	2.38	2.21	1.43	0.32	0.00	0.07	0.09	3.05	3.43	1.62	0.37	11.28
Boleophthalmus pectinirostris	16.27	3.37	3.61	1.02	0.00	0.17	0.64	5.42	7.22	16.71	0.37	45.22
Scartelaos histophorus	7.03	3.04	0.76	0.91	0.00	0.28	0.24	3.13	5.54	22.05	0.68	37.17
Periophthalmodon schlosseri	10.24	4.41	0.78	0.84	0.00	1.07	0.59	2.55	4.86	20.13	0.44	40.46
Periophthalmus magnuspinnatus	12.44	6.94	1.53	0.99	0.00	0.28	0.44	3.89	6.91	17.19	0.41	42.51
Periophthalmus modestus	10.47	6.42	1.44	1.03	0.00	0.75	0.56	5.04	9.65	17.08	0.48	43.81
Oryzias latipes	13.10	8.52	2.51	0.28	0.00	0.10	0.05	1.79	2.81	11.16	0.10	37.41
Kryptolebias marmoratus	11.67	4.33	1.63	0.31	0.00	0.11	0.06	1.67	1.29	7.66	0.13	27.39
Astatotilapia calliptera	16.69	8.96	2.88	0.32	0.00	0.25	0.07	2.07	1.74	3.39	0.08	33.58
Danio rerio	39.44	3.50	5.48	0.51	0.00	0.54	1.00	4.72	5.89	0.37	0.16	55.03
Esox lucius	23.09	6.60	3.70	0.41	0.00	0.34	0.20	2.38	7.28	3.79	0.27	43.49

Table S8: Top 40 GO terms enriched in unique gene families of *P. modestus* where M represents the number of all background proteins, n represents the number of background proteins of a GO term, N represents the number of all test proteins, k represents the number of test proteins of a GO term, P-value represents the Fisher's exact test statistic, and Q-value represents the false discovery rate.

GO ID	M	n	N	k	P-value	Q-value	Namespace	Description
GO:0051253	4645	10	55	2	5.83E-03	3.83E-01	biological process	negative regulation of RNA metabolic process
GO:1903507	4645	10	55	2	5.83E-03	3.83E-01	biological process	negative regulation of nucleic acid-templated transcription
GO:1902679	4645	10	55	2	5.83E-03	3.83E-01	biological process	negative regulation of RNA biosynthetic process
GO:0045892	4645	10	55	2	5.83E-03	3.83E-01	biological process	negative regulation of transcription, DNA-templated
GO:0045934	4645	11	55	2	7.07E-03	3.83E-01	biological process	negative regulation of nucleobase-containing compound metabolic process
GO:0009890	4645	13	55	2	9.88E-03	3.83E-01	biological process	negative regulation of biosynthetic process
GO:0010558	4645	13	55	2	9.88E-03	3.83E-01	biological process	negative regulation of macromolecule biosynthetic process
GO:0031327	4645	13	55	2	9.88E-03	3.83E-01	biological process	negative regulation of cellular biosynthetic process
GO:2000113	4645	13	55	2	9.88E-03	3.83E-01	biological process	negative regulation of cellular macromolecule biosynthetic process
GO:0051301	4645	1	55	1	1.18E-02	3.83E-01	biological process	cell division
GO:0031324	4645	15	55	2	1.31E-02	3.83E-01	biological process	negative regulation of cellular metabolic process
GO:0051172	4645	15	55	2	1.31E-02	3.83E-01	biological process	negative regulation of nitrogen compound metabolic process
GO:0010629	4645	17	55	2	1.67E-02	4.32E-01	biological process	negative regulation of gene expression
GO:0098742	4645	49	55	3	1.97E-02	4.32E-01	biological process	cell-cell adhesion via plasma-membrane adhesion molecules
GO:0098609	4645	49	55	3	1.97E-02	4.32E-01	biological process	cell-cell adhesion
GO:0007156	4645	49	55	3	1.97E-02	4.32E-01	biological process	homophilic cell adhesion via plasma membrane adhesion molecules
GO:0010605	4645	20	55	2	2.28E-02	4.34E-01	biological process	negative regulation of macromolecule metabolic process
GO:0009892	4645	20	55	2	2.28E-02	4.34E-01	biological process	negative regulation of metabolic process
GO:0007059	4645	2	55	1	2.35E-02	4.34E-01	biological process	chromosome segregation
GO:0030036	4645	3	55	1	3.51E-02	5.14E-01	biological process	actin cytoskeleton organization
GO:0030029	4645	3	55	1	3.51E-02	5.14E-01	biological process	actin filament-based process
GO:0016020	2588	622	31	12	4.83E-02	5.14E-01	cellular component	membrane
GO:0004045	7071	4	89	1	4.94E-02	5.14E-01	molecular function	aminoacyl-tRNA hydrolase activity
GO:0051539	7071	4	89	1	4.94E-02	5.14E-01	molecular function	4 iron, 4 sulfur cluster binding
GO:0022610	4645	73	55	3	5.46E-02	5.14E-01	biological process	biological adhesion
GO:0007155	4645	73	55	3	5.46E-02	5.14E-01	biological process	cell adhesion
GO:0009593	4645	5	55	1	5.78E-02	5.14E-01	biological process	detection of chemical stimulus
GO:0034067	4645	5	55	1	5.78E-02	5.14E-01	biological process	protein localization to Golgi apparatus
GO:0000301	4645	5	55	1	5.78E-02	5.14E-01	biological process	retrograde transport, vesicle recycling within Golgi
GO:0051606	4645	5	55	1	5.78E-02	5.14E-01	biological process	detection of stimulus
GO:0000042	4645	5	55	1	5.78E-02	5.14E-01	biological process	protein targeting to Golgi
GO:0072600	4645	5	55	1	5.78E-02	5.14E-01	biological process	establishment of protein localization to Golgi
GO:0050906	4645	5	55	1	5.78E-02	5.14E-01	biological process	detection of stimulus involved in sensory perception
GO:0050907	4645	5	55	1	5.78E-02	5.14E-01	biological process	detection of chemical stimulus involved in sensory perception
GO:0050912	4645	5	55	1	5.78E-02	5.14E-01	biological process	detection of chemical stimulus involved in sensory perception of taste
GO:0030151	7071	5	89	1	6.14E-02	5.14E-01	molecular function	molybdenum ion binding
GO:0019904	7071	5	89	1	6.14E-02	5.14E-01	molecular function	protein domain specific binding
GO:0017025	7071	5	89	1	6.14E-02	5.14E-01	molecular function	TBP-class protein binding
GO:0008527	7071	5	89	1	6.14E-02	5.14E-01	molecular function	taste receptor activity

Table S9: Top 40 GO terms enriched in expanded gene families of *P. modestus* with respect to its common ancestor with *Periophthalmus magnuspinnatus* where M represents the number of all background proteins, n represents the number of background proteins of a GO term, N represents the number of all test proteins, k represents the number of test proteins of a GO term, P-value represents the Fisher's exact test statistic, and Q-value represents the false discovery rate.

GO ID	M	n	N	k	P-value	Q-value	Namespace	Description
GO:0006284	4645	3	63	2	5.38E-04	1.29E-01	biological process	base-excision repair
GO:0007169	4645	7	63	2	3.64E-03	1.29E-01	biological process	transmembrane receptor protein tyrosine kinase signaling pathway
GO:0007167	4645	7	63	2	3.64E-03	1.29E-01	biological process	enzyme linked receptor protein signaling pathway
GO:0032012	4645	7	63	2	3.64E-03	1.29E-01	biological process	regulation of ARF protein signal transduction
GO:0005086	7071	7	109	2	4.7E-03	1.29E-01	molecular function	ARF guanyl-nucleotide exchange factor activity
GO:0004714	7071	7	109	2	4.7E-03	1.29E-01	molecular function	transmembrane receptor protein tyrosine kinase activity
GO:0042623	7071	73	109	5	5.19E-03	1.29E-01	molecular function	ATPase activity, coupled
GO:0015405	7071	54	109	4	9.35E-03	1.29E-01	molecular function	P-P-bond-hydrolysis-driven transmembrane transporter activity
GO:0042626	7071	54	109	4	9.35E-03	1.29E-01	molecular function	ATPase activity, coupled to transmembrane movement of substances
GO:0015399	7071	54	109	4	9.35E-03	1.29E-01	molecular function	primary active transmembrane transporter activity
GO:0043492	7071	54	109	4	9.35E-03	1.29E-01	molecular function	ATPase activity, coupled to movement of substances
GO:0004713	7071	10	109	2	9.77E-03	1.29E-01	molecular function	protein tyrosine kinase activity
GO:0016820	7071	56	109	4	1.06E-02	1.29E-01	molecular function	hydrolase activity, acting on acid anhydrides, catalyzing transmembrane movement of substances
GO:0006020	4645	1	63	1	1.36E-02	1.29E-01	biological process	inositol metabolic process
GO:0009896	4645	1	63	1	1.36E-02	1.29E-01	biological process	positive regulation of catabolic process
GO:0046174	4645	1	63	1	1.36E-02	1.29E-01	biological process	polyol catabolic process
GO:0019310	4645	1	63	1	1.36E-02	1.29E-01	biological process	inositol catabolic process
GO:1901616	4645	1	63	1	1.36E-02	1.29E-01	biological process	organic hydroxy compound catabolic process
GO:0044275	4645	1	63	1	1.36E-02	1.29E-01	biological process	cellular carbohydrate catabolic process
GO:0031023	4645	1	63	1	1.36E-02	1.29E-01	biological process	microtubule organizing center organization
GO:0010508	4645	1	63	1	1.36E-02	1.29E-01	biological process	positive regulation of autophagy
GO:0031331	4645	1	63	1	1.36E-02	1.29E-01	biological process	positive regulation of cellular catabolic process
GO:0046164	4645	1	63	1	1.36E-02	1.29E-01	biological process	alcohol catabolic process
GO:0009052	4645	1	63	1	1.36E-02	1.29E-01	biological process	pentose-phosphate shunt, non-oxidative branch
GO:0005524	7071	673	109	18	1.4E-02	1.29E-01	molecular function	ATP binding
GO:0032559	7071	673	109	18	1.4E-02	1.29E-01	molecular function	adenyl ribonucleotide binding
GO:0019199	7071	12	109	2	1.41E-02	1.29E-01	molecular function	transmembrane receptor protein kinase activity
GO:0030554	7071	674	109	18	1.42E-02	1.29E-01	molecular function	adenyl nucleotide binding
GO:0050113	7071	1	109	1	1.54E-02	1.29E-01	molecular function	inositol oxygenase activity
GO:0005125	7071	1	109	1	1.54E-02	1.29E-01	molecular function	cytokine activity
GO:0016799	7071	1	109	1	1.54E-02	1.29E-01	molecular function	hydrolase activity, hydrolyzing N-glycosyl compounds
GO:0004751	7071	1	109	1	1.54E-02	1.29E-01	molecular function	ribose-5-phosphate isomerase activity
GO:0008430	7071	1	109	1	1.54E-02	1.29E-01	molecular function	selenium binding
GO:0019104	7071	1	109	1	1.54E-02	1.29E-01	molecular function	DNA N-glycosylase activity
GO:0003883	7071	1	109	1	1.54E-02	1.29E-01	molecular function	CTP synthase activity
GO:0003905	7071	1	109	1	1.54E-02	1.29E-01	molecular function	alkylbase DNA N-glycosylase activity
GO:0016798	7071	98	109	5	1.74E-02	1.41E-01	molecular function	hydrolase activity, acting on glycosyl bonds
GO:0019637	4645	113	63	5	1.79E-02	1.42E-01	biological process	organophosphate metabolic process
GO:0032555	7071	852	109	21	1.89E-02	1.42E-01	molecular function	purine ribonucleotide binding
GO:0035639	7071	852	109	21	1.89E-02	1.42E-01	molecular function	purine ribonucleoside triphosphate binding

Table S10: Top 40 GO terms enriched in contracted gene families of *P. modestus* with respect to its common ancestor with *Periophthalmus magnuspinnatus* where M represents the number of all background proteins, n represents the number of background proteins of a GO term, N represents the number of all test proteins, k represents the number of test proteins of a GO term, P-value represents the Fisher's exact test statistic, and Q-value represents the false discovery rate.

GO ID	M	n	N	k	P-value	Q-value	Namespace	Description
GO:0010181	7071	5	32	2	1.97E-04	2.09E-02	molecular function	FMN binding
GO:0043167	7071	1695	32	16	1.21E-03	5.2E-02	molecular function	ion binding
GO:0072593	4645	13	21	2	1.47E-03	5.2E-02	biological process	reactive oxygen species metabolic process
GO:0006432	4645	1	21	1	4.52E-03	7.38E-02	biological process	phenylalanyl-tRNA aminoacylation
GO:0004826	7071	1	32	1	4.53E-03	7.38E-02	molecular function	phenylalanine-tRNA ligase activity
GO:0009378	7071	1	32	1	4.53E-03	7.38E-02	molecular function	four-way junction helicase activity
GO:1903409	4645	2	21	1	9.02E-03	7.38E-02	biological process	reactive oxygen species biosynthetic process
GO:0080134	4645	2	21	1	9.02E-03	7.38E-02	biological process	regulation of response to stress
GO:1901031	4645	2	21	1	9.02E-03	7.38E-02	biological process	regulation of response to reactive oxygen species
GO:0046209	4645	2	21	1	9.02E-03	7.38E-02	biological process	nitric oxide metabolic process
GO:0006809	4645	2	21	1	9.02E-03	7.38E-02	biological process	nitric oxide biosynthetic process
GO:1902882	4645	2	21	1	9.02E-03	7.38E-02	biological process	regulation of response to oxidative stress
GO:0004517	7071	2	32	1	9.03E-03	7.38E-02	molecular function	nitric-oxide synthase activity
GO:0046872	7071	702	32	8	1.1E-02	7.54E-02	molecular function	metal ion binding
GO:0032553	7071	857	32	9	1.14E-02	7.54E-02	molecular function	ribonucleotide binding
GO:0043169	7071	709	32	8	1.17E-02	7.54E-02	molecular function	cation binding
GO:0006000	4645	3	21	1	1.35E-02	7.54E-02	biological process	fructose metabolic process
GO:0016709	7071	3	32	1	1.35E-02	7.54E-02	molecular function	oxidoreductase activity, acting on paired donors, with incorporation or reduction of molecular oxygen, NAD(P)H as one donor, and incorporation of one atom of oxygen
GO:0003873	7071	3	32	1	1.35E-02	7.54E-02	molecular function	6-phosphofructo-2-kinase activity
GO:2001057	4645	4	21	1	1.8E-02	8.69E-02	biological process	reactive nitrogen species metabolic process
GO:0019200	7071	4	32	1	1.8E-02	8.69E-02	molecular function	carbohydrate kinase activity
GO:0008443	7071	4	32	1	1.8E-02	8.69E-02	molecular function	phosphofructokinase activity
GO:0006720	4645	5	21	1	2.24E-02	9.31E-02	biological process	isoprenoid metabolic process
GO:0008299	4645	5	21	1	2.24E-02	9.31E-02	biological process	isoprenoid biosynthetic process
GO:0000166	7071	959	32	9	2.28E-02	9.31E-02	molecular function	nucleotide binding
GO:1901265	7071	959	32	9	2.28E-02	9.31E-02	molecular function	nucleoside phosphate binding
GO:0097367	7071	991	32	9	2.77E-02	9.66E-02	molecular function	carbohydrate derivative binding
GO:0005524	7071	673	32	7	2.8E-02	9.66E-02	molecular function	ATP binding
GO:0032559	7071	673	32	7	2.8E-02	9.66E-02	molecular function	adenyl ribonucleotide binding
GO:0003723	7071	147	32	3	2.81E-02	9.66E-02	molecular function	RNA binding
GO:0030554	7071	674	32	7	2.82E-02	9.66E-02	molecular function	adenyl nucleotide binding
GO:0043168	7071	1007	32	9	3.04E-02	1.01E-01	molecular function	anion binding
GO:0036094	7071	1014	32	9	3.17E-02	1.02E-01	molecular function	small molecule binding
GO:0006281	4645	65	21	2	3.41E-02	1.06E-01	biological process	DNA repair
GO:0033554	4645	68	21	2	3.71E-02	1.09E-01	biological process	cellular response to stress
GO:0006974	4645	68	21	2	3.71E-02	1.09E-01	biological process	cellular response to DNA damage stimulus
GO:0005856	2588	17	6	1	3.88E-02	1.09E-01	cellular component	cytoskeleton
GO:0051716	4645	70	21	2	3.91E-02	1.09E-01	biological process	cellular response to stimulus
GO:0005509	7071	178	32	3	4.56E-02	1.24E-01	molecular function	calcium ion binding
GO:0006801	4645	11	21	1	4.87E-02	1.29E-01	biological process	superoxide metabolic process

Table S11: Statistics of predicted non-coding genes by Infernal (left) and tRNAscan (right).

Type	Number
Cis-reg	312
Ribozyme	3
lncRNA	5
miRNA	455
misc_RNA	10
ncRNA	1
rRNA	2029
sRNA	1
snRNA	370
snoRNA	174
tRNA	1706
vRNA	5
Sum	5071

Type	Number
Ala	97
Arg	102
Asn	65
Asp	60
Cys	25
Gln	104
Glu	80
Gly	161
His	32
Ile	44
Leu	169
Lys	104
Met	100
Phe	41
Pro	49
Pseudo	2823
SeC	4
SeC(e)	1
Ser	159
Sup	1
Thr	110
Trp	35
Tyr	29
Undet	24
Val	91
Sum	4510

Table S12: A list of software and parameters used for genome analyses.

Softwares	Version	Parameters/Commands
JELLYFISH	2.2.6	-C -m 17
GenomeScope	1.0	17_mer_out.histo 17 251 17mer
MiniMAP2	2.13-r86	-t 32
MiniASM	v0.3-r179	default
RACON	1.3.1	-t 32
BWA	0.7.15	mem -t 32
Pilon	1.22	--fix all --mindepth 0.5 --changes --threads 32
tigmint	1.1.2	default
ARCS	1.0.5	default
LINKS	1.8.6	default
HiRise	unrevealed	default
purged_dups	Chinese New Year	-t 32 for minimap2 and default for the others
QUAST	4.5	default
BUSCO	4.1.4	-l metazoa_odb10
trf	4.0.7	2 7 7 80 10 50 500 -f -d -m -h
BuildDatabase	1.0.10	-engine ncbi
RepeatModeler	1.0.10	-engine ncbi -pa 16
RepeatMasker (*de novo*)	4.0.7	-lib library.fa -e ncbi -pa 16 -gccalc -poly -gff
RepeatMasker (RepBase)	4.0.7	-species Fugu -e ncbi -pa 16 -gccalc -poly -gff
LSC	2.0	default
GMAP	2018-07-04	-B 5
gmap2hints.sh	3.3.2	--intronsonly --priority=3 --nomult --ep_cutoff=20
Tophat	2.1.1	--microexon-search --mate-std-dev 26 --mate-inner-dist 38 --min-intron-length 30 --min-coverage-intron 30 --min-segment-intron 30
GenBlastA	1.0.4	-p T -e 1e-5 -g T -f F -a 0.5 -d 100000 -r 100 -c 0.01 -s -100
Exonerate	2.2.0	--model protein2genome --percent 30 --showvulgar no --showalignment yes --showquerygff no --showtargetgff yes --targetchunkid 1 --targetchunktotal 100
BRAKER	2.0	--species=Pmodestus --useexisting --AUGUSTUS_CONFIG_PATH=augustus-3.3.3/config --AUGUSTUS_BIN_PATH=augustus-3.3.2/bin --AUGUSTUS_SCRIPTS_PATH=augustus-3.3.2/scripts --GENEMARK_PATH=gm_et-2019/gmes_petap --cores=48 --AUGUSTUS_ab_initio --gff3 --alternatives-from-evidence=true --softmasking --genome=scaffolds.fa --bam=accepted_hits.bam --hints=full_LR.gff
InterProscan	5.16-55.0	-appl HAMAP,ProDom,PRINTS,Pfam,TIGRFAM,SUPERFAMILY,ProSitePatterns,ProSiteProfiles -goterms -iprlookup
OrthoMCL	2.0.9	-I 1.5
MUSCLE	3.8.31	default
ETE	3.1.1	trimal -gappyout
RAxML	8.2.10	-m PROTGAMMAJTT
MEGA	7.00	megacc
CAFÉ	4.0	default
Inferal	1.1.2	cmscan --rfam --cut_ga --nohmmonly --cpu 10 --tblout infernal.txt --fmt 2 --clanin Rfam.clanin Rfam.cm
RNAmmer	1.2	rnammer -S euk -m lsu,ssu,tsu -xml rnammer.xml -gff rnammer.gff -h rnammer.hmm
tRNAscan	1.3.1	-o trnascan.txt -f trnascan.ss -f trnascan.stat
