# Supplementary material for: Chromosome-level genome assembly of the shuttles hoppfish, Periophthalmus modestus
Source: Gigascience. 2022 Jan 12;11:giab089. doi: 10.1093/gigascience/giab089 (PMC8756193; doi:10.1093/gigascience/giab089)
Supplement: giab089_Response_to_Reviewer_Comments_Revision_2 [file giab089_response_to_reviewer_comments_revision_2.pdf]

Author's Response To Reviewer Comments

Close

The manuscript was revised as requested and confirmed by the editor.

Close
