# Supplementary material for: Chromosome-level genome assembly of the shuttles hoppfish, Periophthalmus modestus
Source: Gigascience. 2022 Jan 12;11:giab089. doi: 10.1093/gigascience/giab089 (PMC8756193; doi:10.1093/gigascience/giab089)
Supplement: giab089_Reviewer_1_Report_Original_Submission — Jiang Hu -- 9/1/2021 Reviewed [file giab089_reviewer_1_report_original_submission.pdf]

Reviewer Report

Title: Chromosome-level genome assembly of the shuttles hoppfish, Periophthalmus modestus

Version: Original Submission **Date:** 9/1/2021

Reviewer name: Jiang Hu

Reviewer Comments to Author:

In this manuscript, Yang et al., presents the genome assembly of the shuttles hoppfish, Periophthalmus modestus. This reference genome was primary assembled using PacBio data and following with a polishing step, then scaffolding using 10X/Hi-C data, and supplemented with annotations via RNA-seq and Iso-seq data. In addition, the author also completed some standard phylogenetic analysis with ~17 Actinopterygii species. The presented results may be a valuable resource for research on shuttles hoppfish and related species. The manuscript is well written. I have few comments that would need to be addressed before publication.
1) The title is about "chromosome-level genome assembly", but the author did not mention how many chromosomes are in the shuttles hoppfish genome and whether the top 23 longest scaffolds are 23 chromosomes?
2) The assembly size (854 Mb) is larger than the estimated haploid genome size (729 Mb), which may be caused by duplicated assembly in heterozygous regions. Authors should use HaploMerger2 (https://github.com/mapleforest/HaploMerger2), or purge_dups (https://github.com/dfguan/purge_dups) to remove one haplotype in highly heterozygous regions, to avoid incorrect downstream analysis, such as comparative genomics.
3) Is there any special reason to use Miniasm for assembly instead of more commonly used assemblers for PacBio data, such as Falcon, Canu.

Level of Interest

Please indicate how interesting you found the manuscript: Choose an item.

Quality of Written English

Please indicate the quality of language in the manuscript: Choose an item.

Declaration of Competing Interests

Please complete a declaration of competing interests, considering the following questions:

- Have you in the past five years received reimbursements, fees, funding, or salary from an organisation that may in any way gain or lose financially from the publication of this manuscript, either now or in the future?

- Do you hold any stocks or shares in an organisation that may in any way gain or lose financially from the publication of this manuscript, either now or in the future?
- Do you hold or are you currently applying for any patents relating to the content of the manuscript?
- Have you received reimbursements, fees, funding, or salary from an organization that holds or has applied for patents relating to the content of the manuscript?
- Do you have any other financial competing interests?
- Do you have any non-financial competing interests in relation to this paper?

If you can answer no to all of the above, write 'I declare that I have no competing interests' below. If your reply is yes to any, please give details below.

I declare that I have no competing interests

I agree to the open peer review policy of the journal. I understand that my name will be included on my report to the authors and, if the manuscript is accepted for publication, my named report including any attachments I upload will be posted on the website along with the authors' responses. I agree for my report to be made available under an Open Access Creative Commons CC-BY license (http://creativecommons.org/licenses/by/4.0/). I understand that any comments which I do not wish to be included in my named report can be included as confidential comments to the editors, which will not be published.

Choose an item.

To further support our reviewers, we have joined with Publons, where you can gain additional credit to further highlight your hard work (see: https://publons.com/journal/530/gigascience). On publication of this paper, your review will be automatically added to Publons, you can then choose whether or not to claim your Publons credit. I understand this statement.

Yes Choose an item.
