# Supplementary material for: Chromosome-level genome assembly of the shuttles hoppfish, Periophthalmus modestus
Source: Gigascience. 2022 Jan 12;11:giab089. doi: 10.1093/gigascience/giab089 (PMC8756193; doi:10.1093/gigascience/giab089)
Supplement: giab089_Reviewer_2_Report_Original_Submission — Yunyun Lv -- 9/7/2021 Reviewed [file giab089_reviewer_2_report_original_submission.pdf]

**Reviewer Report**

**Title: Chromosome-level genome assembly of the shuttles hoppfish, Periophthalmus modestus**

**Version: Original Submission** **Date:** 9/7/2021

**Reviewer name: Yunyun Lv**

**Reviewer Comments to Author:**

The authors completed a chromosome-level assembly of P. modestus. The BUSCOs estimation indicate a relatively high completeness (96.3%) about the genome. The sequencing depth is adequate for the final high-quality genome assembly generation. While the most content of this article clearly present the data and processing, some paragraphs have slight loss of clarity, the details can be as following, which authors need to be revised or rephrased.
Page 4, Line 29-32, Why the RNA preparation here was required to repeat three times ? If it is an approach that test whether contamination is in the extracted materials, why the final three-replicate RNA samples were mixed?
Page 4, Line 39-40, Please revise "To increase genome continuity,"to "To increase continuity in genome assembly"
Page 4, Line 62-64, Please describe more detail about the genome assembling. Miniasm assembling process should be based on minimap2 alignment, Racon is used for contigs polishing with long-reads. The authors should mention these processes with more details. More importantly, authors forget to write how to assemble super-length-scaffolds to chromosome-level scaffolds, this is core for this article, however, it is absent in the method paragraph.
Page 4, Line 73-78, Authors describe "three ways to find repeats", however, i just find two ways, one is to identify tandem repeats and the another is to identify TE.
Page 4, Line 80-81, Authors actually used the RNA-seq based annotated methods according to the following description, but it was not mentioned in the first sentence.
Page 5, Line 24, Change "GO term analysis" to "GO enrichment"
Page 5, Line 99, Are sure the input datasets for gene family clustering is "15 Actinopterygii transcriptomes" or "15 whole-genome gene datasets" ? Usually I used the latter to perform gene families clustering. In addition, authors should describe the standard about how to remove alternative transcript in transcriptomes or whole-genome gene datasets.
Page 6, Line 19, Although this article is aimed to a data-note style, I want to look more result that describe phylogeny and specie divergence time, which benefit readers to understand the academic significance of related to the data generated from this article.

**Level of Interest**

Please indicate how interesting you found the manuscript: Choose an item.

**Quality of Written English**

Please indicate the quality of language in the manuscript: Choose an item.

**Declaration of Competing Interests**

Please complete a declaration of competing interests, considering the following questions:

- Have you in the past five years received reimbursements, fees, funding, or salary from an organisation that may in any way gain or lose financially from the publication of this manuscript, either now or in the future?
- Do you hold any stocks or shares in an organisation that may in any way gain or lose financially from the publication of this manuscript, either now or in the future?
- Do you hold or are you currently applying for any patents relating to the content of the manuscript?
- Have you received reimbursements, fees, funding, or salary from an organization that holds or has applied for patents relating to the content of the manuscript?
- Do you have any other financial competing interests?
- Do you have any non-financial competing interests in relation to this paper?

If you can answer no to all of the above, write 'I declare that I have no competing interests' below. If your reply is yes to any, please give details below.

I declare that I have no competing interests

I agree to the open peer review policy of the journal. I understand that my name will be included on my report to the authors and, if the manuscript is accepted for publication, my named report including any attachments I upload will be posted on the website along with the authors' responses. I agree for my report to be made available under an Open Access Creative Commons CC-BY license (http://creativecommons.org/licenses/by/4.0/). I understand that any comments which I do not wish to be included in my named report can be included as confidential comments to the editors, which will not be published.

Choose an item.

To further support our reviewers, we have joined with Publons, where you can gain additional credit to further highlight your hard work (see: https://publons.com/journal/530/gigascience). On publication of this paper, your review will be automatically added to Publons, you can then choose whether or not to claim your Publons credit. I understand this statement.

Yes Choose an item.
